# Isolation of local strains of the yeast *Metschnikowia* for biocontrol and lipid production purposes

**DOI:** 10.1007/s11274-024-03918-y

**Published:** 2024-02-09

**Authors:** Josep Tatay-Núñez, Joana Albi-Puig, Víctor Garrigós, Margarita Orejas-Suárez, Emilia Matallana, Agustín Aranda

**Affiliations:** 1 Institute for Integrative Systems Biology, CSIC-University of Valencia, I2SysBio. Av. Agustín Escardino 9, Paterna, 46980 Spain; 2 Institute of Agrochemistry and Food Technology, IATA-CSIC, Valencia, Spain

**Keywords:** Active dry yeast, Biocontrol, Biomass, Lipids, *Metschnikowia*, Wine, Yeasts

## Abstract

The bioprospection of indigenous microorganism strains with biotechnological potential represents a prominent trend. *Metschnikowia* yeasts exhibit diverse capabilities, such as ethanol reduction in winemaking, biocontrol potential, and lipid production. In this work, local *Metschnikowia* strains were isolated from different fruits by their ability to produce pulcherrimic acid, a molecule that has been linked to biocontrol activity and that binds iron giving colored colonies. Five strains were selected, each from one of five distinct sources. All of them were identified as *M. pulcherrima*. All five were able inhibit other yeasts and one *M. pulcherrima*, called M7, inhibited the growth of *Aspergillus nidulans*. The selected strains accumulated lipid bodies in stationary phase. Certain non-conventional yeasts like *Hanseniaspora vineae* are very sensitive to biomass drying, but cell extracts from *M. pulcherrima* added to the growth media as a source of antioxidant lipids increased their tolerance to drying. All strains isolated showed good stress tolerance (particularly to heat) and have nutrient requirements similar to a commercial *M. pulcherrima* strain. In addition, the M7 strain had a good growth in sugarcane and beet molasses and behaved like *Saccharomyces cerevisiae* in a growth medium derived from agricultural waste, a persimmon hydrolysate. Therefore, the isolation of local strains of *Metschnikowia* able to grow in a variety of substrates is a good source of biocontrol agents.

**Supplementary Information:**

The online version contains supplementary material available at 10.1007/s11274-024-03918-y.

## Introduction

A current trend in modern bioeconomy focus on the valorization of local resources. Microorganisms are not alien to this trend, and bioprospection to find new or enhanced biotechnological abilities in yeast and bacteria of enological interest is a very promising field. In the traditional practice of winemaking, local yeast varieties are highly valued. The old-fashioned spontaneous fermentation process relies on the microbiota present on grapes and wineries, but in a competitive industry, inoculation with pure starters of selected strains is the usual practice (Pérez-Torrado et al. 2015). This has been done for quite some time with *Saccharomyces cerevisiae* strains, as this is the species that carries out the bulk of alcoholic fermentation. Although wine yeasts can be provided as a refrigerated cream or liquid, due to the stational nature of grape juice fermentations, this starters are usually manufactured as Active Dry Yeasts (ADY), a long-lived format (Pérez-Torrado et al. 2015). The trade-off of a reliable and standardized procedure is the loss of microbial diversity. Non-*Saccharomyces* or non-conventional yeasts are present at the beginning of any wine fermentation as they are overwhelmingly more abundant on the surface of grapes. However, they are displaced by *S. cerevisiae*, inoculated or present in the winery environment, due to its high fermentative power and superior ethanol tolerance. Yet, the initial microbiota impacts on the organoleptic profile of the wine, as those yeasts produce aromas or enzymes that release them from grape components, imprinting their metabolic diversity to the final product (Fleet 2008; Jolly et al. 2014). For this reason, there is a growing interest in the production of selected non-conventional yeasts with relevant properties, although the number of commercially available strains is little compared to *S. cerevisiae* (Roudil et al. 2019; Vejarano and Gil-Calderón 2021). The limitation for using some strains of non-*Saccharomyces* is the difficulty to produce them as dry yeast, due to the harsh environment that implies the loss of 90–95% of cellular water. The mechanisms to deal with stress are different among distantly related yeasts and this affects their potential use as starters (Torrellas et al. 2020). Oxidative stress is the most relevant one during biomass propagation and dehydration (Pérez-Torrado et al. 2015), and those strains sensitive to it can benefit from the addition of external antioxidants to the growth medium, molasses in the case of industrial yeast proliferation (Gamero-Sandemetrio et al. 2015). Vegetable oils, like argan oil, are rich in antioxidants (particularly in unsaturated lipids) and can improve the production of such sensitive strains.

Some of the most promising and versatile non-conventional yeasts belong to the genus *Metschnikowia*. *M. pulcherrima* has many positive roles in winemaking, from release of varietal aromatic compounds to lower volatile acidity and H_2_S reduction in mixed culture fermentations (Morata et al. 2019). It is a fully respiratory yeast that has interest in terms of ethanol reduction. This is very interesting in the current situation of global warming, which implies a rise of fermentable sugars and therefore high alcoholic degree (Orduna 2010). In addition to its impact on wine metabolites, *M. pulcherrima* is known by its ability to refrain the growth of other yeasts and filamentous fungi, and that makes it a suitable biocontrol tool (Sipiczki 2006; Puyo et al. 2023). For this reason, several companies produce starters of this yeast ready for its use in enology (Puyo et al. 2023). Biocontrol can be done via various mechanisms that do not exclude each other, from nutrient depletion to direct interaction, although the most known way is the production of toxins, like the killer factor. Some strains of *Metschnikowia* produce the killer factor. However, the primary and most notable biocontrol mechanism of these yeasts is linked to the generation of pulcherrimic acid, also referred to as pulcherrimin (Sipiczki 2006). This compound possesses chelating properties and is suggested to hinder the pathogen’s access to the iron essential for its survival. This behavior has been proved to be the cause behind the inhibition of the mold that attacks grapes *Botrytis cinerea* in the presence of *M. pulcherrima* (Sipiczki 2006). This phenotype was shared by isolates of the genus *Metschnikowia* that were originally grouped in a clade known as “pulcherrima” consisting of ten species very close genetically (Sipiczki 2020, 2022a). However, due to of the mosaic structures of the genomes, the rDNA repeats do not form continuous arrays and thus cannot be homogenized, so the traditional standard rDNA barcodes can give wrong results and for this reason this clade has been labelled as “fastidious” (Turchetti et al. 2023). Latest advances in genomic research have led to the grouping of all species into the oldest described name, M. pulcherrima (Sipiczki 2022b; Troiano et al. 2023). Outside the enological field, *M. pulcherrima* is also an oleaginous yeast, meaning that it accumulates intracellular lipids when the conditions force it to do so, with high carbon sources and low nitrogen sources. Compared to the other prominent oleaginous yeasts of the genus *Yarrowia* or *Rhodotorula*, *M. pulcherrima* features lower lipid yields (Abeln and Chuck 2021). However, due to its biocontrol properties and tolerance to low pH and temperature, it can be used to produce lipid in non-sterile industrial environments, like open air tank reactors, without any significant contamination (Santamauro et al. 2014). This is interesting in the modern circular bioeconomy, where agricultural and industrial wastes are studied to be used as food stock for microorganisms of biotechnological interest. The high levels of unsaturated fatty acids compared to other non-*Saccharomyces* yeasts has been linked in our laboratory to a high tolerance of this species to dehydration (Torrellas et al. 2020).

In this work autochthonous *Metschnikowia* strains were isolated from local environment sources with two different, but compatible, aims: serving as biocontrol agents and providing an unexpensive source of lipids. The goal was to isolate biocontrol strains better adapted to local conditions. Strains of enological origin were prospected, but other fruits like persimmon and blueberry were also used to increase variety and not to limit its future potential use. These strains could be used to prevent growth of filamentous fungi on the pre- and post-harvest fruits. Inhibition of yeast and molds was indeed found. The other aim was to isolate strains that can be used as a cost-effective source of lipids. Those lipids were used as antioxidant agents to prevent loss of viability during yeast biomass drying. Growth in molasses and agricultural waste as growth substrate was also analyzed.

## Materials and methods

### Yeast isolation and identification

Fruits of different origin (Table 1) were vortexed in sterile centrifuge tubes in the presence of water. Serial dilutions were made and plated. In the case of grape must, dilutions were made directly from fresh juice obtained from tempranillo grapes in Bodegas Pasiego (Sinarcas, Spain). The selective medium used was YGC (Yeast extract 5 g/L, glucose 20 g/L, chloramphenicol 0.1 g/L, sodium propionate 1 g/L, agar 15 g/L) supplemented with 10 mg/L FeCl_3_. Red-colored colonies were isolated in YPD (Yeast extract 10 g/L, glucose 20 g/L, peptone 20 g/L). To extract DNA, colonies were resuspended in 3 µL 0,01 M NaOH, frozen and heated for 10 min at 95 °C. PCR for identification was carried out with oligos ITS1 (5’-TCCGTAGGTGAACCTGCGG-3’) and ITS4 (5’-TCCTCCGCTTATTGATATGC-3’) to amplify ITS (Esteve-Zarzoso et al. 1999) and with oligos NL-1 (5’-GCATAT CAATAAGCGGAGGAAAAG-3’) and NL-4 (5’-GGTCCGTGTTTCAAGACGG-3’) to amplify the D1/D2 domain of 26 rRNA (Kurtzman and Robnett 1998). PCR fragments were purified and sequenced by the Sanger method by the University of Valencia facilities (SCSIE, Burjassot). The sequences were searched using the Megablast program for highly similar sequences in the NCBI BLAST suite (blast.ncbi.nlm.nih.gov) against *Metschnikowia* (taxid:27,320) database. All strains used are described in Supplementary Table S1.

**Table 1:** Isolation of *Metschnikowia* yeasts from fruits in the Valencia province (Spain)

Origin	Location	Code	Number of isolated colonies
Grape must	Sinarcas	M	15
Grapes	Sinarcas	S	11
Passified grapes	Bonrepòs i Mirambell	P	5
Blackberry	Alfara del Patriarca	Z	4
Green persimmon kaki	Moncada	Kv	5
Ripe persimmon kaki	Moncada	Km	5
Senescent persimmon kaki	Moncada	Ks	6
Total			51

### Growth and stress tests

Isolated strains to be tested were grown overnight in YPD, together with reference strains *M. pulcherrima* (whose commercial name is Flavia) and *M. fructicola* of enological origin provided by Lallemand Inc (Canada). Ten-fold serial dilutions in water were made considering the initial OD_600_. Five- µl drops were plated in YPD, and YP + 20 g/L of fructose (YPF), sucrose (YPS), maltose (YPM) and soluble starch (YPSt) to test for carbon sources. Nitrogen was tested in minimal medium SD (1.7 g/L Yeast Nitrogen Base, 20 g/L glucose and 5 g/L ammonium sulfate) or changing the ammonium sulfate by 5 g/L glutamic acid, proline or urea. Stress tolerance was tested by similar spot analysis (Carrasco et al. 2001). To test thermotolerance serial dilutions were plated on YPD plates, that were incubated at different temperatures. To test for ethanol and osmotic tolerance the serial dilutions were spotted on plates containing ethanol (5 and 10%) and 1. 5 M KCl. Those chemicals were added to YPD before pouring the plates. To test for oxidative and acid stress, 100 µL of a dilution of OD_600_ = 1 for each strain, from a stationary culture in YPD medium, were spread on YPD plates. Once the plates were dry, a small filter paper disc was placed in the middle of the plate and 5 µL of hydrogen peroxide or acetic acid was added. Plates were incubated one day and the inhibition halo was measured. The average of three different experiments was plotted.

### Microbial inhibition and pulcherrimic acid production tests

In order to test biocontrol activity against other yeasts a lawn of yeast was made on minimal medium SD using *Candida tropicalis* (Lallemand Inc, Canada) and *Starmerella bacillaris* St8 (Capece et al. 2022) as described for the oxidative stress test. The *Metschnikowia* yeast were grown to saturation in YPD for two days and 5 µL of each strain was placed in the lawn of sensitive strains, plates were incubated for two days and the inhibition halo was measured. Experiments were carried out in triplicate. In a similar way spores from fully sporulated filamentous fungus *Aspergillus nidulans* (FGSCA4) were collected in water, counted microscopically with a hemacytometer and 10^4^ of them were spread in each PDA medium plate.

In order to test pulcherrimic acid production Synthetic Minimal Agar SMA (Horváth et al. 2021) was prepared. It contains NH_4_SO_4_, 5 g/L, MgSO_4_ 0.5 g/L, KH_2_PO_4_ 1 g/L, glucose 20 g/L, agar 25 g/L supplemented with vitamin stock 10 mL/L (biotine 0.5 mg/L, calcium pantotenate 1 mg/L, thiamine 1 mg/L) and FeCl_3_ was added to different concentrations, or to 1 mg/L by default. pH was adjusted to 4,3. Amino acid addition of arginine, leucine and glutamic acid was done at 0.2 g/L. 5 µl of each yeast to test grown to saturation was used to create a megacolony.

### Lipid quantification and lipid body staining with Nile red

Lipid content estimation was conducted using the fluorescent Nile red (9-(diethylamino)-5 H-benzo[a]phenoxazin-5-one) staining method (Ayadi et al. 2018). The fluorescence intensity determines the neutral lipid content and lipid droplet accumulation. The experiments began by cultivating yeast in YPD media overnight, followed by inoculation into high C:N ratio SD minimal medium (25 g/L carbon source, 1.7 g/L yeast nitrogen base, and 0.3 g/L nitrogen source) and grown for three days until saturation. Subsequently, their density was adjusted with Phosphate Buffered Saline (PBS) 1x to OD_600_ = 1 The fluorescence was measured each 5 min during 20 min using a spectrofluorometer (Varioskan Lux plate reader) with the excitation wavelength at 530 nm and emission wavelength at 590 nm. The results were expressed as Relative Fluorescence Units (RFUs), representing the maximum fluorescence recorded. Blank samples were generated with the mixture of reagents used (Nile red diluted in acetone 1 mg/mL, PBS 1x, DMSO diluted in PBS 1:1 (v/v) and substituting the volume of the cell suspension with PBS 1x. Cell autofluorescence was measured using samples that were not treated with Nile red (substituting by acetone). Same staining was performed to visualize fluorescence under a Leica fluorescence microscope. *M. pulcherrima* M7 strain was grown in minimal medium with glucose and ammonium sulfate to be source of lipids. Cells were harvested by centrifugation and washed. Autolysis was conducted using sulfuric acid 0.05% (v/v) and incubation during 60 min at 60ºC. The autolysis product was separated by centrifugation after neutralizing it with Tris-HCl 1 M pH 8 buffer and boiled at 100ºC during 3 min to completely disrupt cells and frozen for subsequent use.

### Biomass propagation and drying

Molasses were prepared by diluting beet and cane molasses extract to achieve a sucrose concentration of 6% (w/v) and supplemented with salts (ammonium sulphate (NH_4_)_2_SO_4_, 0.75% (w/v), monopotassium phosphate KH_2_PO_4_, 0.35% (w/v), magnesium sulphate heptahydrate MgSO_4_·7H_2_O, 0.075% (w/v) and vitamins (biotin, 0.5 mg/L, calcium pantothenate 1 mg/L, thiamine hydrochloride 1 mg/L). The pH of the medium was adjusted to 4.5 using phosphoric acid. The sterilization involved an autoclave process of the diluted molasses and salts separately, while the vitamins were sterilized through filtration using 0.45 μm filters (Gamero-Sandemetrio et al. 2015; Torrellas et al. 2020). Strains from YPD precultures were inoculated to OD_600_ = 0.1 in flasks with 100 mL of molasses and fermentation followed by weight loss and OD_600_. Final biomass was collected by centrifugation, washed with water and weighed. In the case of the dehydration experiments of *H. vineae*, biomass was placed in paper filters and dried in a fluidized dehydrator (Tornado model 501, Sherwood Scientific, United Kingdom), under the parameters of airflow of 2.5 m^3^/min and temperature 37 ºC over a period of 42 min, that leads to a 5% of remaining humidity (Torrellas 2020).

For the valorization of agricultural waste 170 g/L of persimmon pulp or potato was homogenized and hydrolyzed with 1.5% sulfuric acid and heating in autoclave (Ayadi et al. 2018). Then salts were added ((MgSO_4_ 1.5 g/L, CaCl_2_ 0.2 g/L, ZnSO_4_ 0.01 g/L, MnSO_4_ 0.07 g/L, FeSO_4_ 0.007 g/L, CuSO_4_ 0.001 g/L). Then medium was filtered with paper filter, neutralized to pH 4.5, and sterilized by filtration. Growth was performed as done with molasses.

## Results

### Isolation of autochthonous *Metschnikowia* yeasts from agricultural environments

The first step of this work involved the isolation of *Metschnikowia* strains from local fruits, such as grapes (intact and dried), persimmon and blackberry, but also from grape juice from a cellar (Table 1). The addition of ferric chloride in isolation plates helped the identification of red colonies potentially producing pulcherrimin, a desirable trait linked to biocontrol activity. Several colonies with high pigmentation from five locations were chosen and their ITS1-5.8 S rDNA-ITS2 region amplified. All of them gave the expected amplification product of around 400 bp similar to the reference *M. pulcherrima* commercial strain Flavia (data not shown). Next, one isolate from each of the five biological sources (M7, S_1_6, P1, Z4, Km1) was selected for ITS and D1-D2 26 S rDNA regions sequencing (see Materials and Methods section and Supplementary Fig. 1). rDNA barcoding can be difficult for this genus as rDNA repeats are not homogenized (Sipiczki 2020). M7, P1, Z4 and Km1 strains were labelled as *M. pulcherrima*, and although S_1_6 was originally identified as *M. fructicola* by the old phylogeny, it was also considered *M. pulcherrima* accordingly to the latest nomenclature of the clade (Sipiczki 2022b) .

### Physiological characterization of the isolated strains

**Fig. 1:**
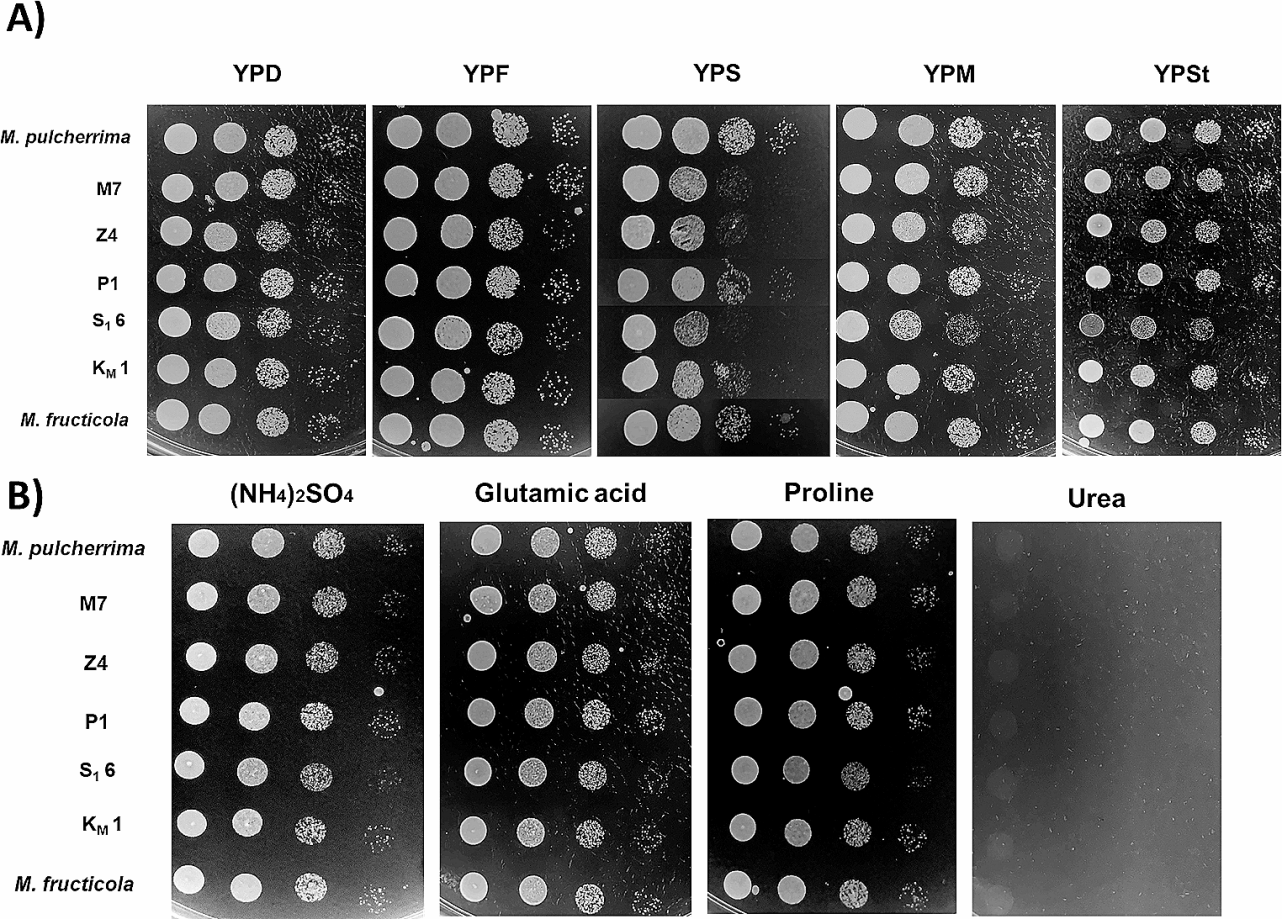
Nutrient requirements of isolated strains. Spot analysis carried out from serial dilutions of stationary phase cultures of each strain in YPD. 5 µl drops of each dilution were plated. **A** Carbon source test. Rich medium containing 2% glucose (YPD), Fructose (YPF), Sucrose (YPS), Maltose (YPM) and starch (YPSt) were tested. **B** Nitrogen source test. Minimal medium SD containing 0.5% of ammonium sulfate, glutamic acid, proline and urea as nitrogen source were tested

First, the five strains selected and identified were tested for metabolic requirements and stress tolerances by spot test analysis. That was done to discard any major growth problem that would prevent their potential industrial use. Figure 1A shows growth in different carbon sources of the five selected strains together with reference strains of enological origin *M. pulcherrima* (Flavia) and *M. fructicola*. As expected, they all grew fine in monosaccharides like glucose and fructose. There was also robust growth in maltose and starch. In this case, S_1_6 showed the slowest growth, even less than the reference *M. fructicola* strain. Sucrose was a suboptimal carbon source for all strains, particularly for M7, Z4 and S_1_6. This was not unexpected, as it is know that *Metschnikowia* does not have a secreted invertase like other yeasts (Torrellas et al. 2023). Regarding the nitrogen source (Fig. 1B), rich nitrogen sources like ammonium and glutamate gave a good amount of growth for all strains, as expected (Conrad et al. 2014). Interestingly, a generally considered poor nitrogen source like proline supported a strong growth in all strains. This is relevant as proline is the most abundant amino acid in grape juice. However, there was no growth in urea, a cheap nitrogen source that can be used as alternative to ammonium, suggesting that none of the strains tested have the enzymatic ability to assimilate it.

**Fig. 2:**
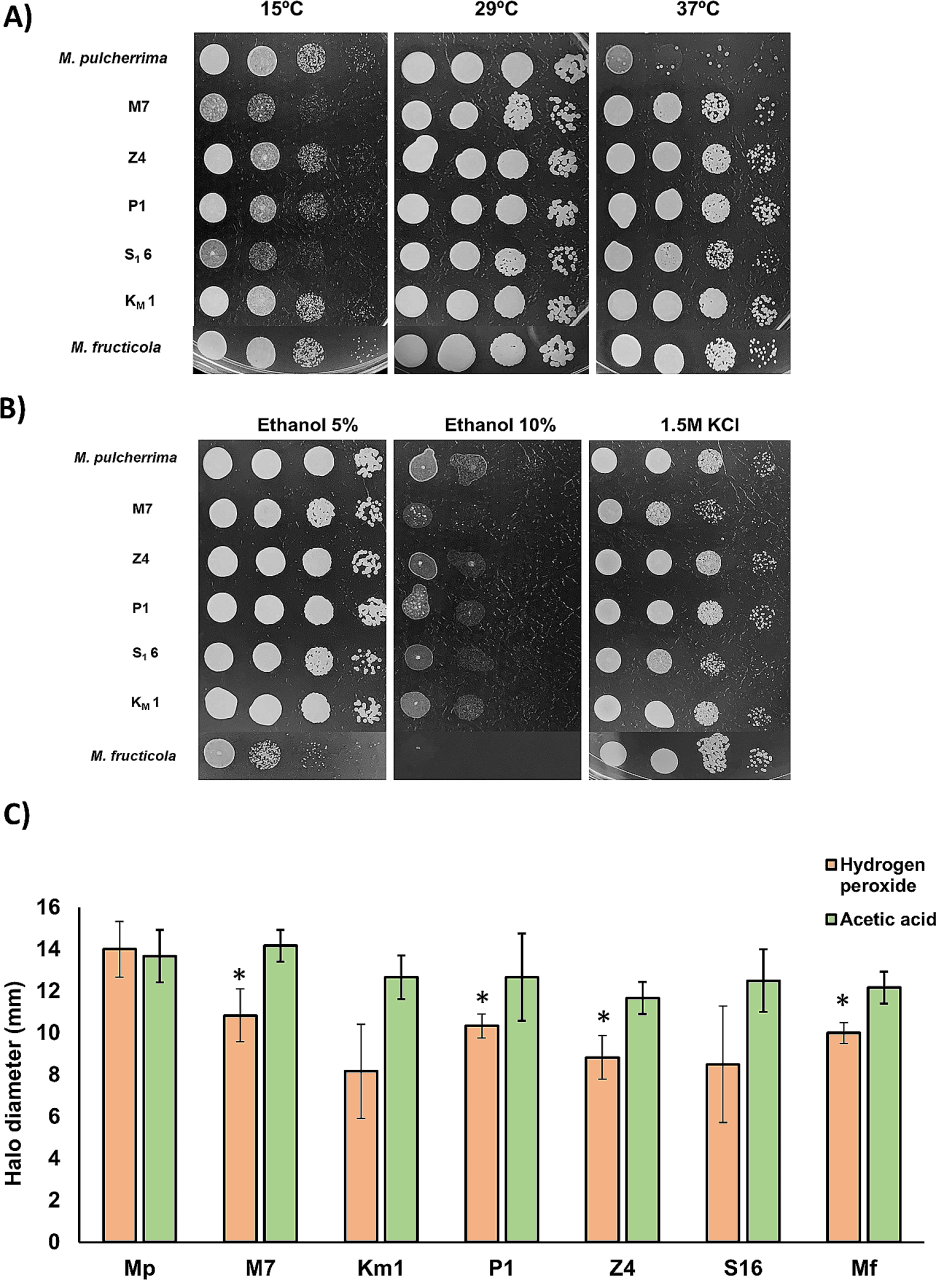
Stress tolerance of isolated strains. Spot analysis carried out from serial dilutions of stationary phase cultures of each strain in YPD. 5 µl of each dilution were plated. **A** Temperature analysis. YPD plates were incubated at 15, 30 and 37 °C. **B** Ethanol and hyperosmotic stress. YPD plates containing 5 and 10% ethanol and 1.5 M KCl were incubated at 30 °C. **C** Oxidative and acid stress. A lawn for each strain to test was made form stationary cultures on YPD plates and paper discs containing 5 µl of 33% H_2_O_2_ or pure acetic acid were placed in the middle and inhibition halo was measured the next day. Experiments were done in triplicate and the average and standard deviation is shown. *, *P* < 0.05, unpaired t test, two-tailed, using *M. pulcherrima* Flavia strain (Mp) as reference

Stress tolerance is a key factor for biotechnological success of yeasts. Several stress tests were performed (Fig. 2). All strains grew well at 29 °C (Fig. 1), so two extreme temperatures were tested (Fig. 2A). Growth is generally reduced at the cooler temperature of 15 °C, particularly for M7 and S_1_6. This temperature is at the lower range for wine fermentation. All isolated strains grew fine at the highest temperature tested, 37 °C. Interestingly they grew much better than the reference *M. pulcherrima* strain Flavia, at the same level that the *M. fructicola* control strain. Therefore, those isolates seem to be better adapted to high temperatures, being isolated from a warm region, and that may potentially give them a biotechnological advance in some processes. Ethanol is a relevant stress in wine fermentation. It is known that *Metschnikowia* does not tolerate the high ethanol levels at the end of fermentation. Indeed, all strains grew poorly at 10% ethanol (Fig. 2B). However, they all coped fine with 5% ethanol, so they are expected to survive during the first days of wine fermentations or in processes that do not accumulate high levels of alcohol. Osmotic stress is a common stress at the beginning of fermentation, so an hyperosmotic shock with1.5 M KCl was tested (Fig. 2B). All strains were able to cope with this stress, being M7 and S_1_6 slightly less tolerant.

Yeasts face toxic molecules during their biotechnological tasks. Oxidative stress is a typical challenge during biomass propagation and dehydration (Pérez-Torrado et al. 2015), and acid tolerance is common in many processes where the biomass have been exposed to acid hydrolysis to release simple sugars. To test oxidative stress, hydrogen peroxide was used, and to test acid tolerance, acetic acid was chosen. As those are volatile and labile molecules, adding them to the hot agar medium is difficult, so an assay consisting of measuring the inhibition halo on a lawn of the yeast of interest was performed (Fig. 2C). Regarding oxidative stress, the inhibition halo of the newly isolated *M. pulcherrima* strains was smaller than the one from reference strain, indicating a better tolerance to this stress. Regarding acetic acid tolerance, all strains behaved in a very similar way, so this seem to be a trait with low variability among natural isolates. Overall, the isolated strains showed a fair stress tolerance, indicating that no major problem would be expected in their biotechnological use.

### Pulcherrimin production and biocontrol potential of the isolated strains

**Fig. 3:**
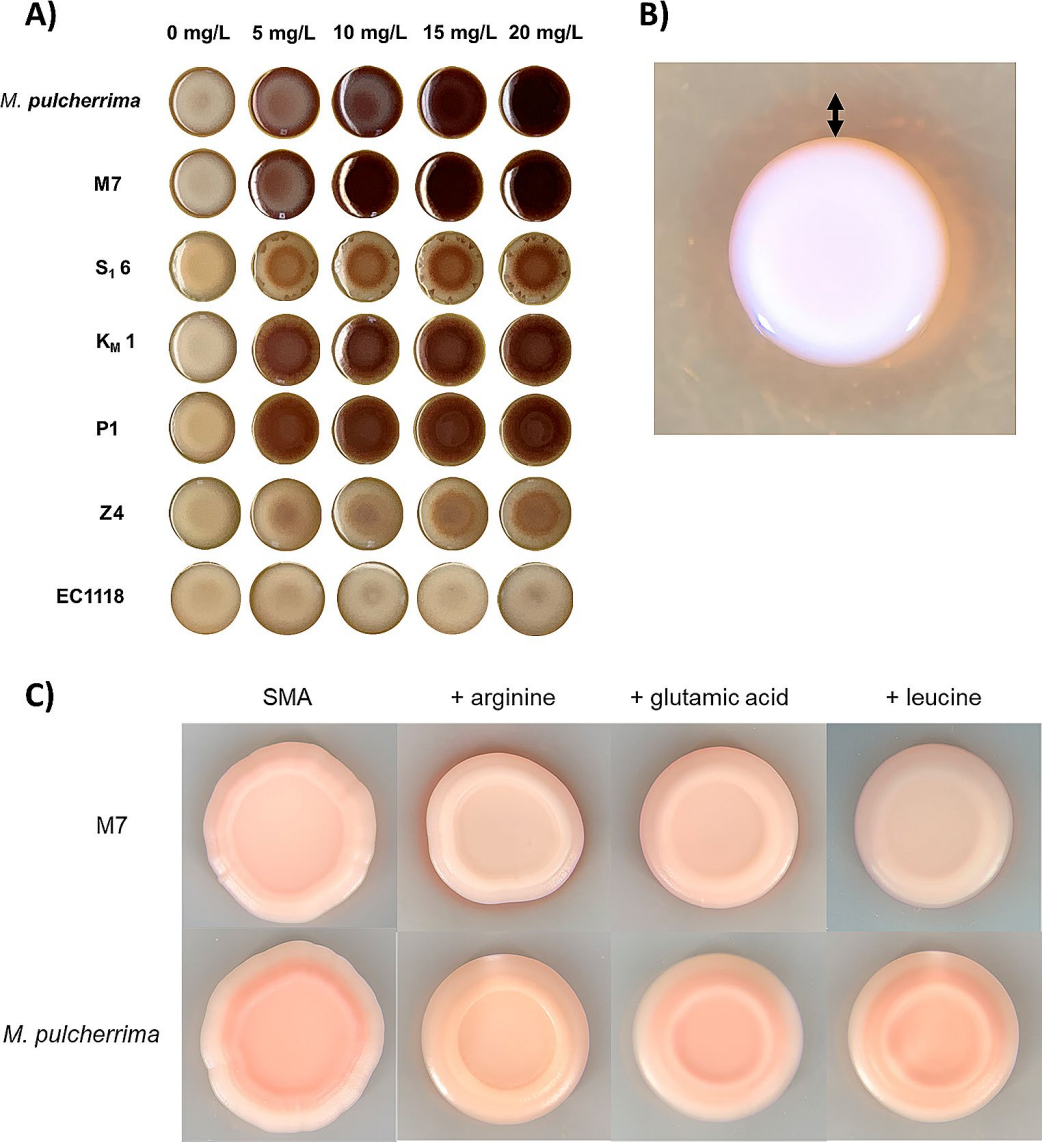
Pulcherrimin production by the isolated strains. **A** Visual analysis under increasing amounts of iron. 5 µl drops of cultures of each strain adjusted to an OD_600_ = 1 were spotted on SMA medium with increasing amounts of FeCl_3_. **B** Picture of M7 strain drop in YPD. The arrow shows the halo formed around the colony. **C** Addition of selected amino acids to SMA medium at 20 mg/L concentration. M7 and reference *M. pulcherrima* Flavia strain were tested

The selected strains were tested for their relative ability to produce pulcherrimin by a visual approach, growing them in increasing amounts of iron. Due to the chelator ability of this pigment a red color is expected (Fig. 3A). All strains showed a reddish color compared to the negative control. *S. cerevisiae* commercial wine strain EC1118, that gives cream colonies. *M. fructicola* S_1_6 and blackberry Z4 are lightly colored, Km1 and P1 are intermediate, and grape must M7 strain shows the strongest pigmentation, even more than the reference *M. pulcherrima* commercial reference strain Flavia, as it reaches full color intensity at a concentration of 10 mg/mL FeCl_3_. It is known that in a medium with no addition of iron, like standard YPD, diffusion of pulcherrimin produces a halo when catching the iron present in the environment of the colony (Sipiczki 2006). This was also the case for the isolated strains (Fig. 3B shows strain M7 as an example). Amino acid abundance in the medium trigger pulcherrimin production by *M. andauensis* (Horváth et al. 2021). We observed the same pattern in our M7 isolate (Fig. 3C), particularly when arginine is present. Therefore, our selected strains produce a good amount of pulcherrimin with the physiological behavior expected.

**Fig. 4:**
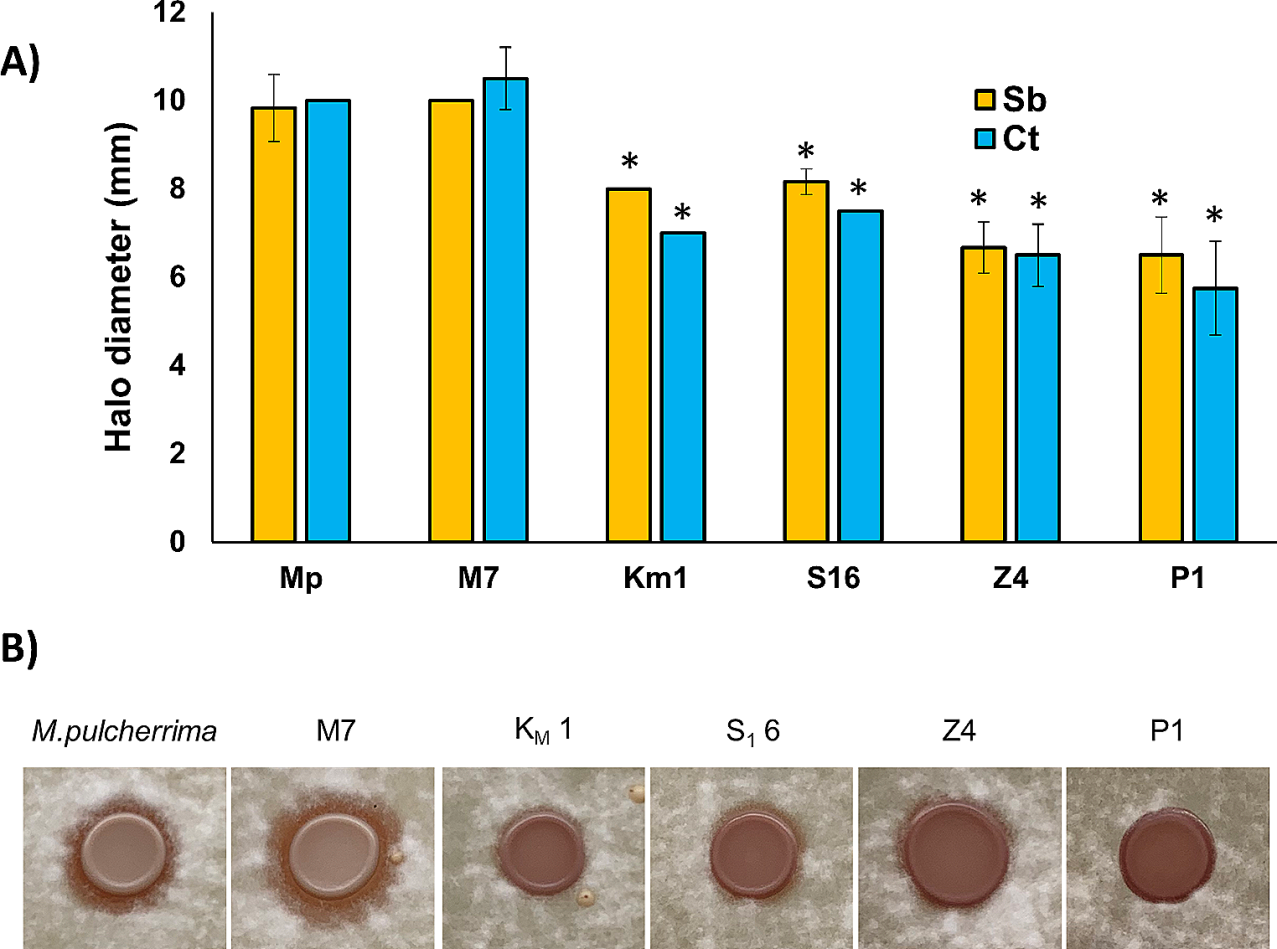
Biocontrol activity of the strains of interest. **A** inhibition halos caused on a lawn of sensitive strains (*Starmerella bacillaris*-St and *Candida tropicalis*-Ct) by a 5 µl drops of cultures of each strain adjusted to an OD_600_ = 1. Experiments were done in triplicate and the average and standard deviation is shown. *, *P* < 0.05, unpaired t test, two-tailed, using *M. pulcherrima* Flavia strain (Mp) as reference. **B** Picture depicting inhibition of the selected strains on plates were conidia of *Aspergillus nidulans* was plated. Picture was taken after 3 days

Next, those isolates were tested by their biocontrol potential. To do so, drops of each selected strains were placed in a lawn of two sensitive yeasts, *Starmerella bacillaris and Candida tropicalis* (Morata et al. 2019), and growth inhibition halos were measured (Fig. 4A). Every isolated strain demonstrated the ability to generate the mentioned halo, whereas *S. cerevisiae* did not exhibit this feature (data not shown). In general, the strains exhibited equal effectiveness against both sensitive yeasts, suggesting a shared biocontrol mechanism. Notably, M7 and the reference *M. pulcherrima* strain exhibited the highest inhibition capacity, while the other isolated strains behaved in a comparable manner. A similar experiment was carried out with a model filamentous fungus, *Aspergillus nidulans* as target (Fig. 4B). In this case most isolated strains were ineffective to prevent the growth of the mycelium of this mold, so a single picture is shown to reflect this fact. However, again M7 produced a clear, yet small, inhibition halo. So did the reference strain, but to a lower extent. Therefore, *M. pulcherrima* M7 it is the best potential candidate for further biocontrol applications.

### Lipid production by the *Metschnikowia* isolated strains

**Fig. 5:**
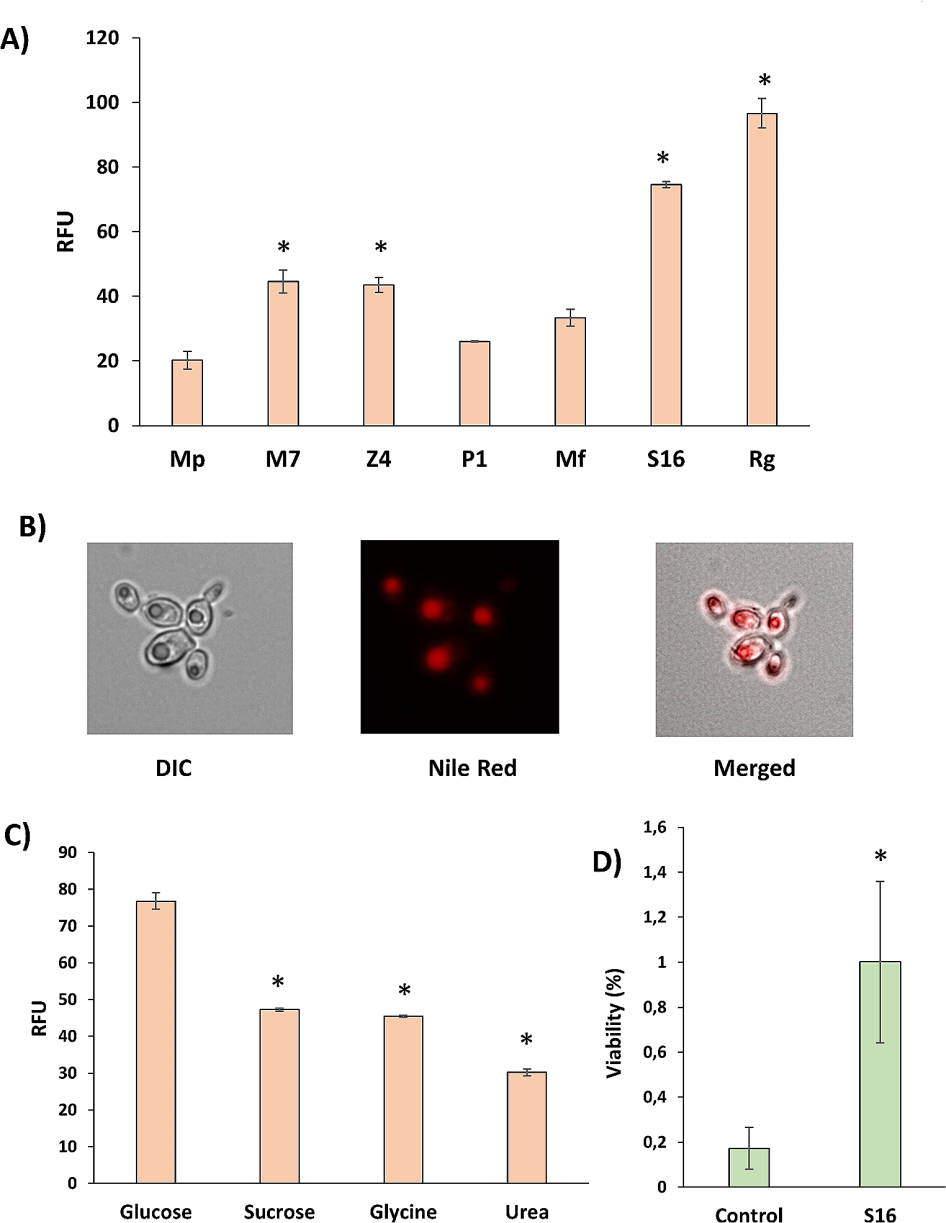
Lipid production by *Metschnikowia* strains. **A** Relative lipid production measured by Nile Red fluorescence (RFU: relative fluorescence units). Cells grown to stationary phase in minimal medium were incubated with Nile Red and fluorescence was measured in a Varioskan plate reader. *Rhodorula graminis* (Rg) was includes as control. Experiments were done in triplicate and the average and standard deviation is shown. *, *P* < 0.05, unpaired t test, two-tailed, using *M. pulcherrima* Flavia strain (Mp) as reference. **B** Fluoresce microscopy of stationary cells of S_1_6 strain incubated with Nile Red. DIC: differential interference contrast. **C** Fluorescence lipid measurement of S_1_6 strain grown with different nutrients. *, *P* < 0.05, unpaired t test, two-tailed, using Glucose + ammonium as control. **D** Viability after biomass drying of *Hanseniaspora vineae* grown in sugar beet molasses without (Control) or with (S16) a lysate of *M. pulcherrima* S_1_6 strain grown in minimal medium with glucose and ammonium sulfate. *, *P* < 0.05, unpaired t test, two-tailed

Next, the ability of these strains to produce lipids was also tested. *Metschnikowia* can act as oleaginous yeast when carbon is plenty and nitrogen limiting. To obtain a relative classification of the strains of interest, their lipid content was measured with Nile red fluorescent probe staining from cultures grown in minimal medium with low nitrogen (Fig. 5). A *Rhodotorula graminis* strain (Rg) isolated from grapes by our laboratory was used as a positive control. Strain Km1 did not grow so efficiently in this medium as the other strains, so it was not included. The *M. pulcherrima* reference strain and P1 isolate had similar levels (Fig. 5a). M7 and Z4 showed the highest level among *M. pulcherrima* isolates, even more than *M. fructicola* reference strain, so there was no clear correlation with the species inside the *Metschnikowia* strains. S_1_6 strain was the strain showing highest fluorescence, although not as big as the *Rhodotorula* strain. Therefore, it was used as model strain for lipid production in the next experiments. Microscopic observation showed the presence of distinctive bodies in all strains in stationary phase in the mentioned medium (Fig. 5B shows S_1_6 strain as example). When stained with Nile red, those structures proved to be indeed lipid bodies, reinforcing the idea that those strains can be used as lipid sources and the simple observation with an optic microscope can be used to follow qualitatively lipid accumulation.

S_1_6 was grown into stationary phase in minimal medium with different sugar and nitrogen composition to test the relative impact of medium composition in the lipid compositions (Fig. 5C). In the presence of ammonium as nitrogen source, glucose and sucrose were tested, being in this case glucose the best carbon source to this effect. In a similar way, using glucose as carbon source, glycine and urea were used as alternative nitrogen sources. However, they do not improve the lipid accumulation that ammonium provides, being urea the poorest nitrogen source. Therefore, a medium with glucose as carbon source and ammonium as nitrogen source is the most promising one for this strain in lipid production.

One aim of this study is to prove the potential use of oleaginous yeasts as providers of antioxidant lipids to improve yeast biomass drying. Purifying lipids is a complex and expensive procedure so as proof of concept we prepared an extract of S_1_6 by autolysis, incubating the biomass produced in the aforementioned medium (glucose and low ammonium) in slightly acidic conditions with heat (see Materials and Methods). This extract was added to the molasses where a non-*Saccharomyces* yeast of enological interest, *Hanseniaspora vineae*, was grown. This yeast was chosen due to its extreme sensibility to the dehydration process used to produce ADY (Torrellas et al. 2020). As expected, viability after drying was very poor (Fig. 5D), but the presence of the extract produced by S_1_6 strain increases viability five-fold. While overall viability remains insufficient for industrial applications, it was proved that lipids produced by yeast can provide protection under adverse conditions.

### Biomass production of *Metschnikowia*

**Fig. 6:**
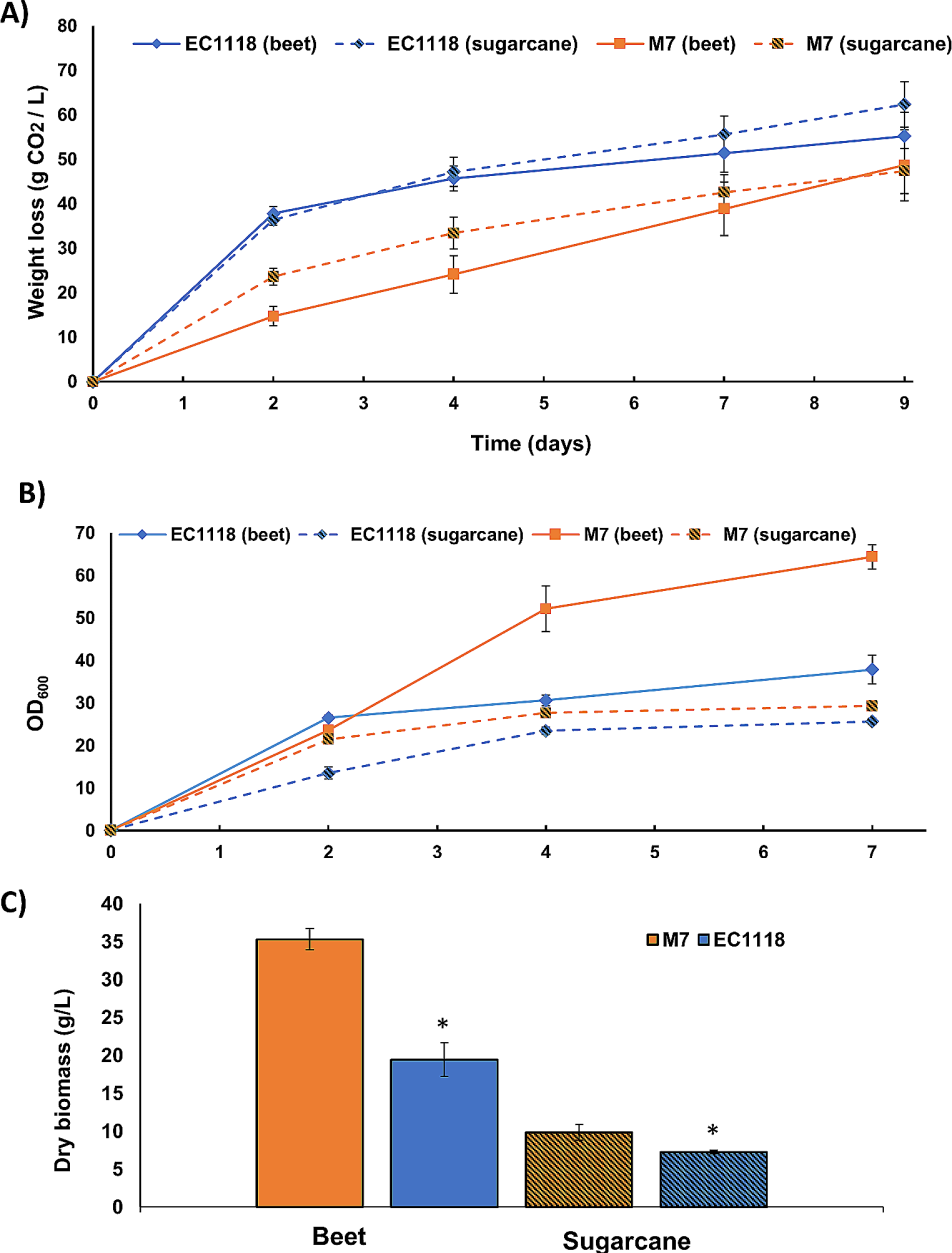
Growth in molasses of M7 strain. **A** Weight loss in beet and cane molasses. M7 *M. pulcherrima* strain and S. cerevisiae *EC1118* strain were compared. **B** Biomass measurement by OD600. Same strains and conditions as panel A. **C** Biomass production at the end of fermentation. The biomass obtained after the experiment described in panels A and B was washed and its weight measured. Experiments were done in triplicate and the average and standard deviation is shown. *, *P* < 0.05, unpaired t test, two-tailed, comparing both strains

The goal of this work was to isolate *Metschnikowia* strains with potential biotechnological applications, like biocontrol potential or lipid production. Once identified and characterized, the next step is to produce their biomass in an efficient and cost-effective way. The next experiments were carried out with M7 strain as example, as it shows the most promising biocontrol potential. The usual way to produce biomass is to propagate yeasts in molasses, a cheap sugar source rich in sucrose. As previously seen (Fig. 2A), sucrose is not the best sugar source for *Metschnikowia* strains growth in laboratory media, probably due to the lack of secreted invertase activity (Torrellas et al. 2023). However, there was growth, so there are alternative pathways in place. First, proliferation was tested for M7and the *S. cerevisiae* wine commercial strain EC1118 as a control (Fig. 6). Two different molasses were used, beet and a sugar cane (the most common sources of table sugar) diluted to have the same level of 6% of sucrose content. The fermentation was followed by weight loss (Fig. 6A) and cell proliferation was measured by optical density at 600 nm (Fig. 6B). Fermentation by EC1118 is strong in both molasses, although at the end sugar cane one reaches a slightly higher level (Fig. 6A). M7 strain is slower in terms of weight loss in both cases and does not reach the levels reached by *S. cerevisiae*, reinforcing the idea that *S. cerevisiae* is a species with higher fermentative power. For M7, fermentation started faster in cane molasses, but both reach similar levels at the end. Regarding optical density (OD; Fig. 6B), the pattern was different. The initial optical densities of both strains were similar, but in this case M7 in beet molasses reached the highest levels. For both strains, cane gave less density than beet molasses. At the end of propagation, biomass was collected, washed and weighed (Fig. 6C) and the results match the final OD values: beet is better substrate, and M7 is produced better than EC1118. The discrepancies with the weight loss profile may indicate that in this particular experiment, *M. pulcherrima* adopts a more efficient respiratory metabolism that produces more biomass per molecule of sucrose consumed. In any case, this novel strain can be produced efficiently in molasses for further application as biocontrol agent.

**Fig. 7:**
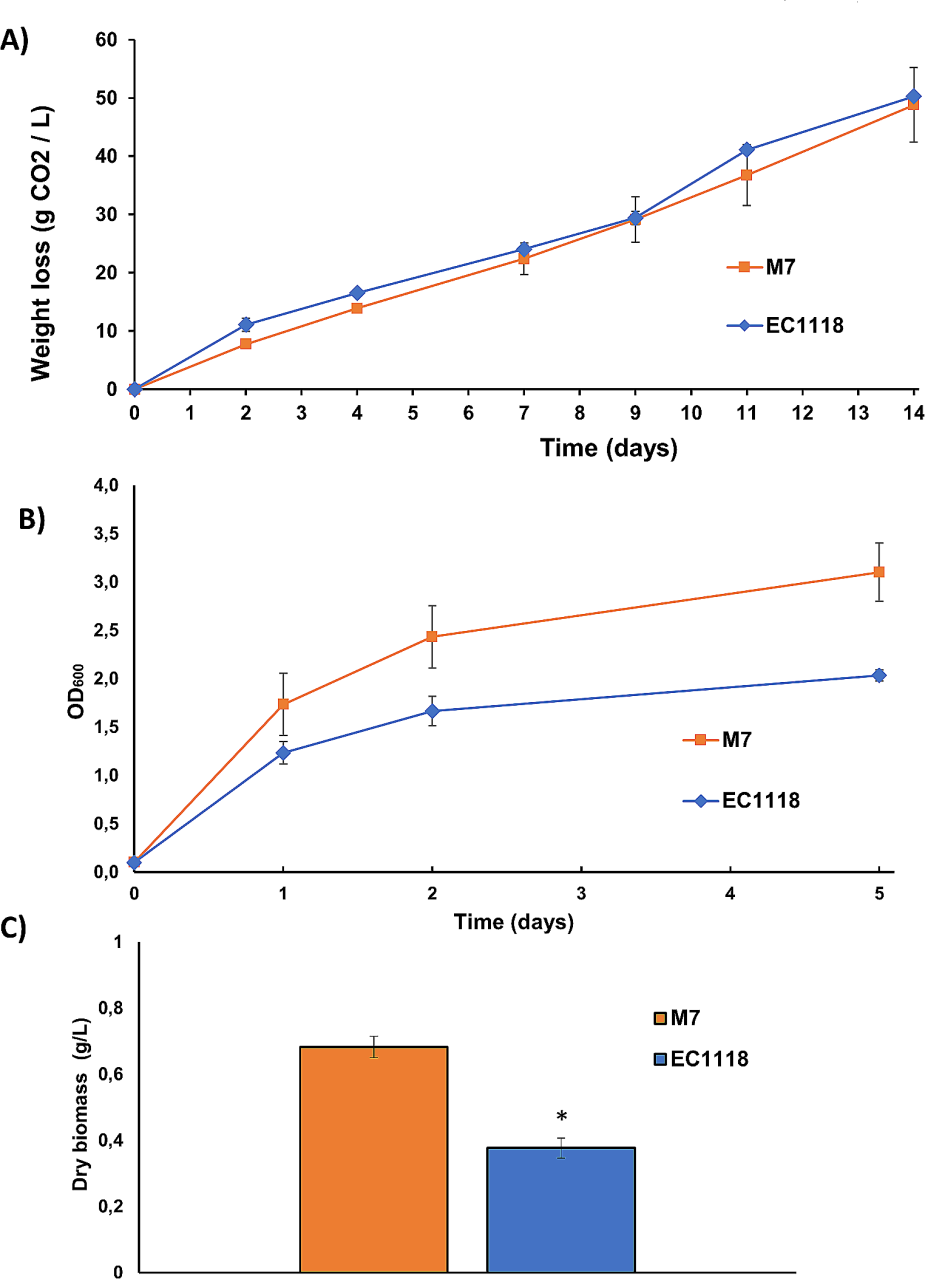
Biomass production of M7 strain in persimmon kaki. **A** Weight loss in kaki hydrolisate. M7 *M. pulcherrima* strain and S. cerevisiae *EC1118* strain were compared. **B** Biomass measurement by OD600. Same strains and conditions as panel A. **C** Biomass production at the end of fermentation. The biomass obtained after the experiment described in panels A and B was washed and its weight measured. Experiments were done in triplicate and the average and standard deviation is shown. *, *P* < 0.05, unpaired t test, two-tailed, comparing both strains

In the current circular bioeconomy, the revalorization of agricultural waste is a very desirable trend. The cultivation of the persimmon kaki produces a big volume of damage fruit so hydrolized persimmon pulp can be a proximity substrate for yeast biomass production. From this fruit a *M. pulcherrima* strain called Km1 was isolated, but M7 strain was used to compare easily with Fig. 6. Weight loss (Fig. 7A), OD_600_ (Fig. 7B) and final biomass (Fig. 7C) were obtained as described before for molasses. Weight loss is similar for both strains (Fig. 7A), while absorbance (Fig. 7B) and final biomass (Fig. 7C) were higher for M7 strain compared to EC1118 (Fig. 7B). This is a very preliminary result, but indicates that *M. pulcherrima* M7 is able to adapt and use a variety of natural substrates in a similar way than a commercial *S. cerevisiae*. In another approach, the lipid producing S_1_6 strain performed similarly to the *S. cerevisiae* control in terms of weight loss in a potato hydrolysate medium (Supplementary Fig. 2), so there is potential for using different substrates in order to propagate autochthonous yeasts diversity found in nature.

## Discussion

Bioprospection for microbial strains with new or improved abilities is a prevailing trend in modern biotechnology. Nature offers a phenotypic variety from which interesting traits for industrial use can be obtained. In this work local *Metschnikowia* strains were isolated from agricultural environments with two aims: finding new biocontrol agents and a source of lipids that can be used as antioxidants to improve Active Dry Yeast (ADY) production. The screening was based in the color produced by the antimicrobial molecule pulcherrimic acid in the presence of iron (Sipiczki 2006), so the primary filter was for strains with potential biocontrol capacity. Indeed, the isolated strain with stronger color, *M. pulcherrima* M7 (Fig. 3), was the one with better inhibition potential against other yeasts and the only one to inhibit a filamentous fungus (Fig. 4). Therefore, it seems that the biocontrol potential may be caused by pulcherrimin production. This is also a desired ability for the potential lipid-producing strains, as its ability to inhibit microbial growth may come useful when used in non-sterile substrates like agricultural waste (Santamauro et al. 2014). However, it is known that other oleaginous yeasts like *Yarrowia* and *Rhodotorula* produce a better yield of lipids (Abeln and Chuck 2021), as seen when comparing the *Metschnikowia* strains with a *R. graminis* of enological origin (Fig. 5A). Therefore, additional comparative analysis in biotechnological conditions are required to choose the better option. Strain S_1_6 was the most promising one in terms of lipid production potential, and although it does not produce the strongest color in the presence of iron nor inhibits *A. nidulans*, it is able to suppress growth of the two yeasts tested (Fig. 4). These tests were done with target species from our laboratory collections and like the reference *Metschnikowia* strains are of enological origin. Those strains have been selected for their suitability for winemaking and probably are not the most effective ones against plagues, but they are better adapted to grape and cellar environments. In the future a more thorough analysis using pathogenic strains present in the field would be interesting. It is possible that *Metschnikowia* is better adapted to compete with the local microbiota, and a better inhibition can be obtained choosing the right biocontrol agent-pest pair. In any case, strains with broad inhibition spectrum are also of commercial interest. Overall, the isolated strains have a robust growth in several media (Fig. 1) and have a good stress tolerance (Fig. 2), similar to the reference strains. The *M. pulcherrima* strains behave similarly to the commercial strain Flavia. Interestingly, all the isolated strains have a higher tolerance to heat than the reference strain Flavia, that is the better adapted to 15 °C. This strain was isolated in Chile (Acuña-Fontecilla et al. 2017), a very different geographical location. Therefore, these particular local strains from Mediterranean origin seem to be better adapted to higher temperatures, so they may be better fitted for certain applications. For instance, if the strains are to be used to prevent damage in grapes and other products by spraying them on the surface of the fruit during the summer months, thermal tolerance could give those strains an edge.

To be effective as a biocontrol agent (or eventually as lipid producer), substantial yeast biomass has to be generated. The traditional way to produce *S. cerevisiae* involves growth in molasses, from sugar beet or cane, in conditions of high oxygenation and low sugar to prevent the Crabtree effect and impose respiration (Pérez-Torrado et al. 2015). *Metschnikowia* is a Crabtree-negative genus (Barkhuizen et al. 2021), so this problem does not exist. However, sucrose is the worst carbohydrate to support growth among the ones tested (Fig. 1). It has been described that *M. pulcherrima* does not show a secreted invertase activity like *S. cerevisiae* (Schnierda et al. 2014; Torrellas et al. 2023). Indeed genome sequencing (Gore-Lloyd et al. 2019) failed to find an ortholog of *S. cerevisiae SUC2* gene, but there are alpha-glucosidases/glucoamylases annotated that may contribute to intracellular hydrolysis. That may explain the fact that, although *M. pulcherrima* M7 strain grows slower than *S. cerevisiae* in molasses (Fig. 6), the final yield is better, probably due to the fact that all carbon is going through respiration and there is not a fraction channeled to fermentation, as that may be the case for *S. cerevisiae* in this flask approach.

As the number of biotechnological interesting yeasts and their applications increase, the demand for growth substrates not suitable for human food production will increase. The valorization of local agricultural waste is a trend that will help to achieve a more sustainable bioeconomy (Singh and Singh 2022). The use this kind of waste to produce yeast biomass has not been fully exploited. For instance, in the province where the strains were isolated a growing persimmon production takes place. A preliminary approach using a persimmon hydrolysate indicates that *M. pulcherrima* M7 strain behaves even better than commercial *S. cerevisiae* strain in terms of biomass production in this substrate (Fig. 7). The yields are not as high as the ones produced in molasses, but indicates that *Metschnikowia* strains have a robust growth in several substrates, overall as good as commercial *S. cerevisiae*. The fact that those strains use efficiently an amino acid such as proline, very abundant in grape juice, and that is considered poor for *S. cerevisiae*, open the possibility to use batches of grape must not optimal for winemaking for the proliferation of those yeasts.

Previous studies in our laboratory indicate that selected strains of *M. pulcherrima* and *M. fructicola* have a strong tolerance to dehydration (Torrellas et al. 2020). Our isolated strains have a similar stress tolerance, so no problem to produce them as ADY is expected. This tolerance to desiccation can come handy to use them as a preemptive bioprotection agent that could be sprayed on the surface of fruits and allowed to dry. *Metschnikowia* strains were relatively tolerant to dehydration compared to *S. cerevisiae*, that it is usually more tolerant that most of non-*Saccharomyces* yeasts of enological interest. For instance, *Hanseniaspora vineae* is much more labile than other yeasts of enological origin (Torrellas et al. 2020). One of the causes of drying tolerance was related to the lipid composition of their membranes. *M. pulcherrima* has polyunsaturated fatty acids, while *S. cerevisiae* only have monounsaturated, and *H. vineae* had lower levels oleic acid (Torrellas et al. 2020). Exogenous antioxidant lipids coming from argan oil have been proved to increase viability after drying of sensitive *S. cerevisiae* strains (Gamero-Sandemetrio et al. 2015). It also helps to increase viability of *H. vineae* to drying (Marcó 2020). Therefore, the use of *Metschnikowia* biomass can be a source of useful unsaturated fatty acids to prevent drying damage in *H. vineae*. That was seen using autolysates of S_1_6 strain (Fig. 5D). S_1_6 was the strain that relatively produced more lipids compared to the other ones (Fig. 5A). The initial screening was carried out searching for pulcherrimin production, so a more specific screening could be done in the future in order to select for the most promising strains. In any case a simple microscopic visualization in nitrogen-limiting conditions showed the presence of lipid bodies in this and all the other *Metschnikowia* strains (Fig. 5B and data not shown), so the lipid assimilation can be found and followed easily in any industrial environment. We cannot rule out that other components than lipids may be helping the viability of *H. vineae*. A purification of each component would be necessary for validation of the protective role of lipids, but additional purification steps at industrial level would not be as cost efficient as a simple autolysis. Maybe a fractionation of those lipid bodies or co-culture between sensitive and tolerant strains of enological interest can be alternatives to be studied in the future. In conclusion, *Metschnikowia* isolates can be a useful source of lipids to enhance dry biomass production using molasses and eventually from non-sterile agricultural waste.

### Electronic supplementary material

Below is the link to the electronic supplementary material.

Supplementary Material 1

Supplementary Material 2

Supplementary Material 3

## Data Availability

The data underlying this article are available in the article and in its online supplementary material.

## Materials availability

All strains are available upon request.
