# Supplementary material for: Isolation of local strains of the yeast Metschnikowia for biocontrol and lipid production purposes
Source: World J Microbiol Biotechnol. 2024 Feb 9;40(3):88. doi: 10.1007/s11274-024-03918-y (PMC10857958; doi:10.1007/s11274-024-03918-y)
Supplement: Supplementary file 1 — Supplementary Material 1 [file 11274_2024_3918_MOESM1_ESM.docx]

| Strain code | Species | Origin | Commercial name |
| --- | --- | --- | --- |
| EC1118 | *Saccharomyces cerevisiae* | Lallemand | Lalvin EC1118 |
| Mp | *Metschnikowia pulcherrima* | Lallemand | Flavia |
| Mf | *Metschnikowia fructivola* | Lallemand |  |
| Hv | *Hanseniaspora vineae* | Lallemand |  |
| Ct | *Candida tropicalis* | Lallemand |  |
| Sb | *Starmerella bacillaris St8* | Angela Capece laboratory |  |
| Rg | *Rhodothorula graminis* | Laboratory collection |  |
| M7 | *Metschnikowia pulcherrima* | This work, grape juice |  |
| P1 | *Metschnikowia pulcherrima* | This work, dry grape |  |
| Km1 | *Metschnikowia pulcherrima* | This work, persimmon |  |
| Z4 | *Metschnikowia pulcherrima* | This work, blackberry |  |
| S_1_6 | *Metschnikowia fructivola* | This work, grape |  |

**Supplemental Table S1. Yeast strains used in this work.**
