# Supplementary material for: Isolation of local strains of the yeast Metschnikowia for biocontrol and lipid production purposes
Source: World J Microbiol Biotechnol. 2024 Feb 9;40(3):88. doi: 10.1007/s11274-024-03918-y (PMC10857958; doi:10.1007/s11274-024-03918-y)
Supplement: Supplementary file 2 — Supplementary Material 2 [file 11274_2024_3918_MOESM2_ESM.pdf]

Supplementary Figure 1.

Molecular identification of the isolated strains. Sequences from the D1/D2 domains of the LSU rRNA genes were used in the Megablast program for highly similar sequences in the BLAST suite of National Center for Biotechnology Information (blast.ncbi.nlm.nih.gov) against *Metschnikowia* (taxid:27320) database. Best 10 hits are shown, and alignment to reference strains is included. Due to problems in the D1/D2 amplification, strain P1 was identified using ITS1-5.8S-ITS2 segments of the rDNA repeats against the type species fungal database.

M7 strain

D1/D2 domain sequence, partial. GenBank PP175919

>M7

GCGGCAAAAGCTCAAATTTGAAATCCCCCGGGAATTGTAATTTGAAGAGATTTGGGTCCGGCCGGCAG
GGGTTAAGTCCACTGGAAAGTGGCGCCACAGAGGGTGACAGCCCCGTGAACCCCTTCAAAGCCTTCAT
CCCAGATCTCCAAGAGTCGAGTTGTTTGGGAATGCAGCTCTAAGTGGGTGGTAAATTCCATCTAAAGCT
AAATACCGGCGAGAGACCGATAGCGAACAAGTACAGTGATGGAAAGATGAAAAGCACTTTGAAAAGA
GAGTGAAAAAGTACGTGAAATTGTTGAAAGGGAAGGGCTTGCAAGCAGACACTTAACTGGGCCAGCA
TCGGGGCGGCGGGGAGCAAAACCACCGGGGAATGTACCTTTCGAGGATTATAACCCCGGCCCTTACTC
CCATACTGCCCCGAGGCCTGCAATCTAAGGATGCTGGCGTAATGGTTGCAAGTCGCCCGTCTTGAAACA
CGGACC

Megablast alignment against Metschnikowia (taxid:27320), first 10 hits

	Description	Scientific Name	Max Score	Total Score	Query Cover	E value	Per. Ident	Acc. Len	Accession
☑	Metschnikowia pulcherrima isolate 3Y138B large subunit ribosomal RNA gene, partial sequence	Metschnikowia pulcherrima	880	880	100%	0.0	99.59%	786	MT225539.1
☑	Metschnikowia pulcherrima isolate 3Y138A large subunit ribosomal RNA gene, partial sequence	Metschnikowia pulcherrima	880	880	100%	0.0	99.59%	779	MT225531.1
☑	Metschnikowia pulcherrima strain FB002 26S ribosomal RNA gene, partial sequence	Metschnikowia pulcherrima	880	880	100%	0.0	99.59%	507	EF139135.1
☑	Metschnikowia pulcherrima strain CEC RCF-0-39 26S ribosomal RNA gene, partial sequence	Metschnikowia pulcherrima	874	874	100%	0.0	99.38%	544	JX103182.1
☑	Metschnikowia pulcherrima strain D77_2 26S ribosomal RNA gene, partial sequence	Metschnikowia pulcherrima	874	874	100%	0.0	99.38%	492	HM627131.1
☑	Metschnikowia sp. (in: Fungi) isolate 1631 large subunit ribosomal RNA gene, partial sequence	Metschnikowia sp. (in: budding ye...	870	870	100%	0.0	99.17%	511	MW405494.1
☑	Metschnikowia pulcherrima isolate Soil1 large subunit ribosomal RNA gene, partial sequence	Metschnikowia pulcherrima	869	869	100%	0.0	99.17%	514	MG707676.1
☑	Metschnikowia pulcherrima culture CBS:2243 large subunit ribosomal RNA gene, partial sequence	Metschnikowia pulcherrima	869	869	100%	0.0	99.17%	833	KY108490.1
☑	Metschnikowia sp. (in: Fungi) 28S ribosomal RNA gene, partial sequence	Metschnikowia sp. (in: budding ye...	869	869	100%	0.0	99.17%	488	KU350434.1
☑	Metschnikowia pulcherrima isolate UTAD201 large subunit ribosomal RNA gene, partial sequence	Metschnikowia pulcherrima	869	869	100%	0.0	99.17%	514	OQ304846.1

Alignment to reference strain *Metschnikowia pulcherrima* culture CBS:5833 large subunit ribosomal RNA gene, partial sequence (ID: KY108497.1)

```
Score         Expect    Identities     Gaps            Strand

863 bits(467)  0.0      477/482(99%)   0/482(0%)       Plus/Plus

Query  1     GCGGCAAAAGCTCAAATTTGAAATCCCCCGGGAATTGTAATTTGAAGAGATTTGGGTCCG  60
             ||||||||||||||||||||||||||||||||||||||||||||||||||||||||||||
Sbjct  134   GCGGCAAAAGCTCAAATTTGAAATCCCCCGGGAATTGTAATTTGAAGAGATTTGGGTCCG  193

Query  61    GCCGGCAGGGGTTAAGTCCACTGGAAAGTGGCGCCACAGAGGGTGACAGCCCCGTGAACC  120
             |||||| |||||||||||||||||||||||||||||||||||||||||||||||||||||
Sbjct  194   GCCGGCGGGGGTTAAGTCCACTGGAAAGTGGCGCCACAGAGGGTGACAGCCCCGTGAACC  253

Query  121   CCTTCAAAGCCTTCATCCCAGATCTCCAAGAGTCGAGTTGTTTGGGAATGCAGCTCTAAG  180
             |||| |||||| ||||||||||||||||||||||||||||||||||||||||||||||||
Sbjct  254   CCTTTAAAGCCCTCATCCCAGATCTCCAAGAGTCGAGTTGTTTGGGAATGCAGCTCTAAG  313
```

```
Query  181   TGGGTGGTAAATTCCATCTAAAGCTAAATACCGGCGAGAGACCGATAGCGAACAAGTACA  240
             ||||||||||||||||||||||||||||||||||||||||||||||||||||||||||||
Sbjct  314   TGGGTGGTAAATTCCATCTAAAGCTAAATACCGGCGAGAGACCGATAGCGAACAAGTACA  373

Query  241   GTGATGGAAAGATGAAAAGCACTTTGAAAAGAGAGTGAAAAAGTACGTGAAATTGTTGAA  300
             ||||||||||||||||||||||||||||||||||||||||||||||||||||||||||||
Sbjct  374   GTGATGGAAAGATGAAAAGCACTTTGAAAAGAGAGTGAAAAAGTACGTGAAATTGTTGAA  433

Query  301   AGGGAAGGGCTTGCAAGCAGACACTTAACTGGGCCAGCATCGGGGCGGCGGGGAGCAAAA  360
             ||||||||||||||||||||||||||||||||||||||||||||||||||||||||||||
Sbjct  434   AGGGAAGGGCTTGCAAGCAGACACTTAACTGGGCCAGCATCGGGGCGGCGGGGAGCAAAA  493

Query  361   CCACCGGGGAATGTACCTTTCGAGGATTATAACCCCGGCCCTTACTCCCATACTGCCCCG  420
             |||||||||||||||||||||||||||||||||||||||||||||||||||||  |||||
Sbjct  494   CCACCGGGGAATGTACCTTTCGAGGATTATAACCCCGGCCCTTACTCCCATACCACCCCG  553

Query  421   AGGCCTGCAATCTAAGGATGCTGGCGTAATGGTTGCAAGTCGCCCGTCTTGAAACACGGA  480
             ||||||||||||||||||||||||||||||||||||||||||||||||||||||||||||
Sbjct  554   AGGCCTGCAATCTAAGGATGCTGGCGTAATGGTTGCAAGTCGCCCGTCTTGAAACACGGA  613

Query  481   CC  482
             ||
Sbjct  614   CC  615
```

S_16 strain

D1/D2 domain sequence, partial. GenBank PP175930

GCGAGTGAGCGGCAAAAGCTCAAATTTGAAATCCCCCGGGAATTGTAATTTGAAGAGATTTGGGTCCG
GCCGGCGGGGGTTAAGTCCACTGGAAAGTGGCGCCACAGAGGGTGACAGCCCCGTGAACCCCTTTAA
CGCCCTCATCCCAGATCTCCAAGAGTCGAGTTGTTTGGGAATGCAGCTCTAAGTGGGTGGTAAATTCCA
TCTAAAGCTAAATACCGGCGAGAGACCGATAGCGAACAAGTACAGTGATGGAAAGATGAAAAGCACT
TTGAAAAGAGAGTGAAAAAGTACGTGAAATTGTTGAAAGGGAAGGGCTTGCAAGCAGACACTTAACT
GGGCCAGCATCGGGGCGGCGGGAAACAAAACCACCGGGGAATGTACCTTTCGAGGATTATAACCCCG
GTCTCAATTTCCTTGTTGCCCCGAGGCCTGCAATCTAAGGATGCTGGCGTAATGGTTGCAAGTCGCCCG
TCTTGAAACACGGA

Megablast alignment against Metschnikowia (taxid:27320), first 10 hits

	Description	Scientific Name	Max Score	Total Score	Query Cover	E value	Per. Ident	Acc. Len	Accession
☑	Metschnikowia fructicola strain UMY553 26S ribosomal RNA gene, partial sequence	Metschnikowia fructicola	896	896	100%	0.0	99.80%	513	KP171590.1
☑	Metschnikowia cf. pulcherrima isolate B14P-2 large subunit ribosomal RNA gene, partial sequence	Metschnikowia cf. pulcherrima	896	896	100%	0.0	99.80%	541	MT749253.1
☑	Metschnikowia aff. fructicola F-b-10 26S ribosomal RNA gene, partial sequence	Metschnikowia aff. fructicola F-b...	896	896	100%	0.0	99.80%	498	FJ842082.1
☑	Metschnikowia sinensis isolate 6-2L large subunit ribosomal RNA gene, partial sequence	Metschnikowia sinensis	891	891	100%	0.0	99.59%	1050	MN123984.1
☑	Metschnikowia pulcherrima isolate UTAD193 large subunit ribosomal RNA gene, partial sequence	Metschnikowia pulcherrima	891	891	100%	0.0	99.59%	518	OQ304841.1
☑	Metschnikowia pulcherrima isolate UTAD10 large subunit ribosomal RNA gene, partial sequence	Metschnikowia pulcherrima	891	891	100%	0.0	99.59%	518	OQ304691.1
☑	Metschnikowia sinensis strain PC-6-49.1 large subunit ribosomal RNA gene, partial sequence	Metschnikowia sinensis	891	891	100%	0.0	99.59%	502	MZ185366.1
☑	Metschnikowia fructicola strain PC-6-12.1 large subunit ribosomal RNA gene, partial sequence	Metschnikowia fructicola	891	891	100%	0.0	99.59%	503	MZ185360.1
☑	Metschnikowia fructicola strain YzA 26S ribosomal RNA gene, partial sequence	Metschnikowia fructicola	891	891	100%	0.0	99.59%	512	HM191666.1
☑	Metschnikowia sinensis isolate 3L2 large subunit ribosomal RNA gene, partial sequence	Metschnikowia sinensis	889	889	100%	0.0	99.59%	970	MN069593.1

Alignment to reference strain *Metschnikowia fructicola* 277 (unitig_213, whole genome shotgun sequence. Sequence ID: ANFW02000056.1).

Alignment statistics for match #1

```
 Score                   Expect     Identities        Gaps          Strand
 891 bits(482)           0.0        487/489(99%)      1/489(0%)     Plus/Minus
Query  1         GCGAGTG-AGCGGCAAAAGCTCAAATTTGAAATCCCCCGGGAATTGTAATTTGAAGAGAT  59
```

```
                 ||||||| ||||||||||||||||||||||||||||||||||||||||||||||||||||
Sbjct  371643  GCGAGTGAAGCGGCAAAAGCTCAAATTTGAAATCCCCCGGGAATTGTAATTTGAAGAGAT  371584

Query  60      TTGGGTCCGGCCGGCGGGGGTTAAGTCCACTGGAAAGTGGCGCCACAGAGGGTGACAGCC  119
               ||||||||||||||||||||||||||||||||||||||||||||||||||||||||||||
Sbjct  371583  TTGGGTCCGGCCGGCGGGGGTTAAGTCCACTGGAAAGTGGCGCCACAGAGGGTGACAGCC  371524

Query  120     CCGTGAACCCCTTTAACGCCCTCATCCCAGATCTCCAAGAGTCGAGTTGTTTGGGAATGC  179
               ||||||||||||||||||||||||||||||||||||||||||||||||||||||||||||
Sbjct  371523  CCGTGAACCCCTTTAACGCCCTCATCCCAGATCTCCAAGAGTCGAGTTGTTTGGGAATGC  371464

Query  180     AGCTCTAAGTGGGTGGTAAATTCCATCTAAAGCTAAATACCGGCGAGAGACCGATAGCGA  239
               ||||||||||||||||||||||||||||||||||||||||||||||||||||||||||||
Sbjct  371463  AGCTCTAAGTGGGTGGTAAATTCCATCTAAAGCTAAATACCGGCGAGAGACCGATAGCGA  371404

Query  240     ACAAGTACAGTGATGGAAAGATGAAAAGCACTTTGAAAAGAGAGTGAAAAAGTACGTGAA  299
               ||||||||||||||||||||||||||||||||||||||||||||||||||||||||||||
Sbjct  371403  ACAAGTACAGTGATGGAAAGATGAAAAGCACTTTGAAAAGAGAGTGAAAAAGTACGTGAA  371344

Query  300     ATTGTTGAAAGGGAAGGGCTTGCAAGCAGACACTTAACTGGGCCAGCATCGGGGCGGCGG  359
               ||||||||||||||||||||||||||||||||||||||||||||||||||||||||||||
Sbjct  371343  ATTGTTGAAAGGGAAGGGCTTGCAAGCAGACACTTAACTGGGCCAGCATCGGGGCGGCGG  371284

Query  360     GAAACAAAACCACCGGGGAATGTACCTTTCGAGGATTATAACCCCGGTCTCAATTTCCTT  419
               ||||||||||||||||||||||||||||||||||||||||||||||||||||||||||||
Sbjct  371283  GAAACAAAACCACCGGGGAATGTACCTTTCGAGGATTATAACCCCGGTCTCAATTTCCTT  371224

Query  420     GTTGCCCCGAGGCCTGCAATCTAAGGATGCTGGCGTAATGGTTGCAAGTCGCCCGTCTTG  479
               |||||||||||||||||| |||||||||||||||||||||||||||||||||||||||||
Sbjct  371223  GTTGCCCCGAGGCCTGCATTCTAAGGATGCTGGCGTAATGGTTGCAAGTCGCCCGTCTTG  371164

Query  480     AAACACGGA  488
               |||||||||
Sbjct  371163  AAACACGGA  371155
```

Km1 strain

D1/D2 domain sequence, partial. GenBank PP175942

GCGGCAAAAGCTCAAATTTGAAATCCCCCGGGAATTGTAATTTGAAGAGATTTGGGTCCGGCCGGCAG
GGGTTAAGTCCACTGGAAAGTGGCGCCACAGAGGGTGACAGCCCCGTGAACCCCTCAACGCCCTCAT
CCCAGATCTCCAAGAGTCGAGTTGTTTGGGAATGCAGCTCTAGTGGGTGGTAAATTCCATCTAAAGCTA
AATACCGGCGAGAGACCGATAGCGAACAAGTACAGTGATGGAAAGATGAAAAGCACTTTGAAAAGAG
AGTGAAAAAGTACGTGAAATTGTTGAAAGGGAAGGGCTTGCAAGCAGACACTTAACTGGGCCAGCAT
CGGGGCGGCGGGGAGCAAAACCACCGGGGAATGTACCTTTCGAGGATTATAACCCCGGCCCTTACTCC
CATACCGCCCCGAGGCCTGCAATCTAAGGATGCTGGCGTAATGGTTGCAAGTCGCCCGTCTTGAA

Megablast alignment against *Metschnikowia* (taxid:27320), first 10 hits

	Description	Scientific Name	Max Score	Total Score	Query Cover	E value	Per. Ident	Acc. Len	Accession
☑	Metschnikowia aff. pulcherrima P01A016 small subunit ribosomal RNA gene, partial sequence; internal transcribe...	Metschnikowia af...	861	861	100%	0.0	99.58%	1203	JX188181.1
☑	Metschnikowia pulcherrima strain D77_2 26S ribosomal RNA gene, partial sequence	Metschnikowia p...	861	861	100%	0.0	99.58%	492	HM627131.1
☑	Metschnikowia pulcherrima isolate UTAD139 large subunit ribosomal RNA gene, partial sequence	Metschnikowia p...	856	856	100%	0.0	99.37%	504	OQ304729.1
☑	Metschnikowia aff. pulcherrima isolate B11 large subunit ribosomal RNA gene, partial sequence	Metschnikowia af...	856	856	100%	0.0	99.37%	484	MN817289.1
☑	Metschnikowia sp. (in: budding yeasts) 26S ribosomal RNA gene, partial sequence	Metschnikowia s...	854	854	99%	0.0	99.57%	516	KT922963.1
☑	Metschnikowia sp. 11-578 clone b51 26S ribosomal RNA gene, partial sequence	Metschnikowia s...	854	854	99%	0.0	99.57%	516	KM249369.1
☑	Metschnikowia sp. (in: budding yeasts) 26S ribosomal RNA gene, partial sequence	Metschnikowia s...	850	850	100%	0.0	99.15%	495	KT922680.1
☑	Metschnikowia pulcherrima isolate BWC12-3Ay large subunit ribosomal RNA gene, partial sequence	Metschnikowia p...	850	850	100%	0.0	99.15%	814	MT039420.1
☑	Metschnikowia aff. pulcherrima isolate D2 large subunit ribosomal RNA gene, partial sequence	Metschnikowia af...	850	850	100%	0.0	99.15%	490	MN817293.1
☑	Metschnikowia pulcherrima isolate Qch4 26S ribosomal RNA gene, partial sequence	Metschnikowia p...	850	850	100%	0.0	99.15%	508	HM067868.1

Alignment to reference strain *Metschnikowia pulcherrima* culture CBS:5833 large subunit ribosomal RNA gene, partial sequence (ID: KY108497.1).

```
Score           Expect  Identities     Gaps          Strand
839 bits(454)   0.0     467/473(99%)   1/473(0%)     Plus/Plus

Query  1    GCGGCAAAAGCTCAAATTTGAAATCCCCCGGGAATTGTAATTTGAAGAGATTTGGGTCCG  60
            ||||||||||||||||||||||||||||||||||||||||||||||||||||||||||||
Sbjct  134  GCGGCAAAAGCTCAAATTTGAAATCCCCCGGGAATTGTAATTTGAAGAGATTTGGGTCCG  193

Query  61   GCCGGCAGGGGTTAAGTCCACTGGAAAGTGGCGCCACAGAGGGTGACAGCCCCGTGAACC  120
            |||||| |||||||||||||||||||||||||||||||||||||||||||||||||||||
Sbjct  194  GCCGGCGGGGGTTAAGTCCACTGGAAAGTGGCGCCACAGAGGGTGACAGCCCCGTGAACC  253

Query  121  CCCTCAACGCCCTCATCCCAGATCTCCAAGAGTCGAGTTGTTTGGGAATGCAGCTCT-AG  179
            || | || ||||||||||||||||||||||||||||||||||||||||||||||||| ||
Sbjct  254  CCTTTAAAGCCCTCATCCCAGATCTCCAAGAGTCGAGTTGTTTGGGAATGCAGCTCTAAG  313

Query  180  TGGGTGGTAAATTCCATCTAAAGCTAAATACCGGCGAGAGACCGATAGCGAACAAGTACA  239
            ||||||||||||||||||||||||||||||||||||||||||||||||||||||||||||
Sbjct  314  TGGGTGGTAAATTCCATCTAAAGCTAAATACCGGCGAGAGACCGATAGCGAACAAGTACA  373

Query  240  GTGATGGAAAGATGAAAAGCACTTTGAAAAGAGAGTGAAAAAGTACGTGAAATTGTTGAA  299
            ||||||||||||||||||||||||||||||||||||||||||||||||||||||||||||
Sbjct  374  GTGATGGAAAGATGAAAAGCACTTTGAAAAGAGAGTGAAAAAGTACGTGAAATTGTTGAA  433

Query  300  AGGGAAGGGCTTGCAAGCAGACACTTAACTGGGCCAGCATCGGGGCGGCGGGGAGCAAAA  359
            ||||||||||||||||||||||||||||||||||||||||||||||||||||||||||||
Sbjct  434  AGGGAAGGGCTTGCAAGCAGACACTTAACTGGGCCAGCATCGGGGCGGCGGGGAGCAAAA  493

Query  360  CCACCGGGGAATGTACCTTTCGAGGATTATAACCCCGGCCCTTACTCCCATACCGCCCCG  419
            |||||||||||||||||||||||||||||||||||||||||||||||||||||| |||||
Sbjct  494  CCACCGGGGAATGTACCTTTCGAGGATTATAACCCCGGCCCTTACTCCCATACCACCCCG  553

Query  420  AGGCCTGCAATCTAAGGATGCTGGCGTAATGGTTGCAAGTCGCCCGTCTTGAA  472
            |||||||||||||||||||||||||||||||||||||||||||||||||||||
Sbjct  554  AGGCCTGCAATCTAAGGATGCTGGCGTAATGGTTGCAAGTCGCCCGTCTTGAA  606
```

Z4 strain

D1/D2 domain sequence, partial. GenBank PP176174

ATGCCTCAGTAACGGCGAGTGAGCGGCAAAAGCTCAAATTTGAAATCCCCCGGGAATTGTAATTTGAA
GAGATTTGGGTCCGGCCGGCAGGGGTTAAGTCCACTGGAAAGTGGCGCCACAGAGGGTGACAGCCCC
GTGAACCCCTTTAACGCCCTCATCCCAGATCTCCAAGAGTCGAGTTGTTTGGGAATGCAGCTCTAAGTG
GGTGGTAAATTCCATCTAAAGCTAAATACCGGCGAGAGACCGATAGCGAACAAGTACAGTGATGGAAA
GATGAAAAGCACTTTGAAAAGAGAGTGAAAAAGTACGTGAAATTGTTGAAAGGGAAGGGCTTGCAAG
CAGACACTTAACTGGGCCAGCATCGGGGCGGCGGGAAACAAAACCACCGGGGAATGTACCTTTCGAG
GATTATAACCCCGGCCCTTACTCCCTTGCCACCCCGAGGCCTGCAATCTAAGGATGCTGGCGTAATGGT
TGCAAGTCGCCCGTCTTGAAACACGGACC

Megablast alignment against Metschnikowia (taxid:27320), first 10 hits

	Description	Scientific Name	Max Score	Total Score	Query Cover	E value	Per. Ident	Acc. Len	Accession
☑	Metschnikowia pulcherrima isolate YQY_A20_26S large subunit ribosomal RNA gene, partial sequence	Metschnikowia p...	902	902	100%	0.0	99.01%	512	OP643941.1
☑	Metschnikowia pulcherrima strain M53 26S ribosomal RNA gene, partial sequence	Metschnikowia p...	902	902	99%	0.0	99.01%	513	GU080051.1
☑	Metschnikowia pulcherrima isolate UTAD182 large subunit ribosomal RNA gene, partial sequence	Metschnikowia p...	900	900	100%	0.0	98.82%	518	OQ304834.1
☑	Metschnikowia pulcherrima strain M320 26S ribosomal RNA gene, partial sequence	Metschnikowia p...	896	896	99%	0.0	98.81%	513	GU478324.1
☑	Metschnikowia pulcherrima isolate CABM7C large subunit ribosomal RNA gene, partial sequence	Metschnikowia p...	891	891	98%	0.0	99.00%	664	MF783068.1
☑	Metschnikowia pulcherrima culture CBS:5833 large subunit ribosomal RNA gene, partial sequence	Metschnikowia p...	891	891	99%	0.0	98.61%	693	KY108497.1
☑	Metschnikowia aff. pulcherrima P01A016 small subunit ribosomal RNA gene, partial sequence; internal transcribe...	Metschnikowia af...	891	891	99%	0.0	98.61%	1203	JX188181.1
☑	Metschnikowia pulcherrima strain NRRL Y-7111 small subunit ribosomal RNA gene, partial sequence; internal tran...	Metschnikowia p...	891	891	99%	0.0	98.61%	1179	JX188179.1
☑	Metschnikowia pulcherrima strain XJ-73 large subunit ribosomal RNA gene, partial sequence	Metschnikowia p...	889	889	99%	0.0	98.61%	513	MN371967.1
☑	Metschnikowia sp. (in: budding yeasts) 26S ribosomal RNA gene, partial sequence	Metschnikowia s...	889	889	98%	0.0	98.99%	537	KT922942.1

Alignment to reference strain *Metschnikowia pulcherrima* culture CBS:5833 large subunit ribosomal RNA gene, partial sequence (ID: KY108497.1).

```
Score           Expect  Identities     Gaps           Strand
891 bits(482)   0.0     497/504(99%)   1/504(0%)      Plus/Plus

Query  2     TGCCTCAGTAACGGCGAGTG-AGCGGCAAAAGCTCAAATTTGAAATCCCCCGGGAATTGT  60
             |||||||||||||||||||| |||||||||||||||||||||||||||||||||||||||
Sbjct  112   TGCCTCAGTAACGGCGAGTGAAGCGGCAAAAGCTCAAATTTGAAATCCCCCGGGAATTGT  171

Query  61    AATTTGAAGAGATTTGGGTCCGGCCGGCAGGGGTTAAGTCCACTGGAAAGTGGCGCCACA  120
             |||||||||||||||||||||||||||| |||||||||||||||||||||||||||||||
Sbjct  172   AATTTGAAGAGATTTGGGTCCGGCCGGCGGGGGTTAAGTCCACTGGAAAGTGGCGCCACA  231

Query  121   GAGGGTGACAGCCCCGTGAACCCCTTTAACGCCCTCATCCCAGATCTCCAAGAGTCGAGT  180
             ||||||||||||||||||||||||||||| ||||||||||||||||||||||||||||||
Sbjct  232   GAGGGTGACAGCCCCGTGAACCCCTTTAAAGCCCTCATCCCAGATCTCCAAGAGTCGAGT  291

Query  181   TGTTTGGGAATGCAGCTCTAAGTGGGTGGTAAATTCCATCTAAAGCTAAATACCGGCGAG  240
             ||||||||||||||||||||||||||||||||||||||||||||||||||||||||||||
Sbjct  292   TGTTTGGGAATGCAGCTCTAAGTGGGTGGTAAATTCCATCTAAAGCTAAATACCGGCGAG  351

Query  241   AGACCGATAGCGAACAAGTACAGTGATGGAAAGATGAAAAGCACTTTGAAAAGAGAGTGA  300
             ||||||||||||||||||||||||||||||||||||||||||||||||||||||||||||
Sbjct  352   AGACCGATAGCGAACAAGTACAGTGATGGAAAGATGAAAAGCACTTTGAAAAGAGAGTGA  411

Query  301   AAAAGTACGTGAAATTGTTGAAAGGGAAGGGCTTGCAAGCAGACACTTAACTGGGCCAGC  360
             ||||||||||||||||||||||||||||||||||||||||||||||||||||||||||||
Sbjct  412   AAAAGTACGTGAAATTGTTGAAAGGGAAGGGCTTGCAAGCAGACACTTAACTGGGCCAGC  471

Query  361   ATCGGGGCGGCGGGAAACAAAACCACCGGGGAATGTACCTTTCGAGGATTATAACCCCGG  420
             |||||||||||||| | |||||||||||||||||||||||||||||||||||||||||||
Sbjct  472   ATCGGGGCGGCGGGGAGCAAAACCACCGGGGAATGTACCTTTCGAGGATTATAACCCCGG  531

Query  421   CCCTTACTCCCTTGCCACCCCGAGGCCTGCAATCTAAGGATGCTGGCGTAATGGTTGCAA  480
             ||||||||||| | ||||||||||||||||||||||||||||||||||||||||||||||
Sbjct  532   CCCTTACTCCCATACCACCCCGAGGCCTGCAATCTAAGGATGCTGGCGTAATGGTTGCAA  591

Query  481   GTCGCCCGTCTTGAAACACGGACC  504
             ||||||||||||||||||||||||
Sbjct  592   GTCGCCCGTCTTGAAACACGGACC  615
```

<u>P1 strain</u>

ITS sequence. GenBank PP176176

TCCGTAGGTGAACCTGCGGAAGGATCATTAATATTGTTATTACACCCTTTTAGGCACAAACTCTAAATCT
TTACCGTCAATAACTCAATCAAAAAACTTTCAACAACGGATCTCTTGGTTCTCGCATCGATGAAGAACGC
AGCGAATTGCGATACGTAATATGACTTGCAGACGTGAATCATTGAATCTTTGAACGCACATTGCGCCCC
GGGGTATTCCCCAGGGCATGCGTGGGTGAGCGATATTTACTCTCAAACCTCCGGTTTGGTCCTGCTTCG
GCCTAATATCAACGGCGCTAGAATAAGTTTTAGCCCCATTCTTCTTCCTCACCCTCGTAAGACTACCCGC
TGAACTTAAGCATATCAATAAGCGGAGGA

Megablast alignment against fungal ITSs, type strains database (rRNA_typestrains/

ITS_RefSeq_Fungi).

	Description	Scientific Name	Max Score	Total Score	Query Cover	E value	Per. Ident	Acc. Len	Accession
☑	Metschnikowia pulcherrima NRRL Y-7111 ITS region; from TYPE material	Metschnikowia pulcherrima	658	658	100%	0.0	98.14%	434	NR_164379.1
☑	Metschnikowia ziziphicola CBS 10358 ITS region; from TYPE material	Metschnikowia ziziphicola	658	658	99%	0.0	98.40%	387	NR_166218.1
☑	Metschnikowia sinensis CBS 10357 ITS region; from TYPE material	Metschnikowia sinensis	580	580	99%	4e-166	94.67%	390	NR_155404.1
☑	Metschnikowia shanxiensis CBS 10359 ITS region; from TYPE material	Metschnikowia shanxiensis	580	580	99%	4e-166	94.67%	388	NR_155403.1
☑	Metschnikowia chrysoperlae ATCC MYA-4304 ITS region; from TYPE material	Metschnikowia chrysoperlae	566	566	100%	1e-161	93.68%	381	NR_111376.1
☑	Metschnikowia pimensis ATCC MYA-4306 ITS region; from TYPE material	Metschnikowia pimensis	560	560	100%	5e-160	93.42%	381	NR_111377.1
☑	Metschnikowia vanudenii CBS 9134 ITS region; from TYPE material	Metschnikowia vanudenii	438	438	100%	2e-123	87.76%	540	NR_155405.1
☑	Metschnikowia chrysomelidarum ATCC MYA-4344 ITS region; from TYPE material	Metschnikowia chrysomelidarum	436	436	100%	9e-123	87.56%	386	NR_111396.1
☑	Metschnikowia rancensis CBS 8174T ITS region; from TYPE material	Metschnikowia rancensis	431	431	100%	4e-121	87.43%	392	NR_155369.1
☑	Metschnikowia cibodasensis UICC Y-335 ITS region; from TYPE material	Metschnikowia cibodasensis	394	394	98%	5e-110	85.82%	389	NR_138172.1

Alignment to Metschnikowia pulcherrima NRRL Y-7111 ITS region; from TYPE material (Sequence ID: NR_164379.1).

```
Score            Expect  Identities     Gaps          Strand
658 bits(356)    0.0     370/377(98%)   0/377(0%)     Plus/Plus

Query  1    TCCGTAGGTGAACCTGCGGAAGGATCATTAATATTGTTATTACACCCTTTTAGGCACAAA  60
            |||||||||||||||||||||||||||||||||||||| |||||||||||||||||||||
Sbjct  17   TCCGTAGGTGAACCTGCGGAAGGATCATTAATATTGTTTTTACACCCTTTTAGGCACAAA  76

Query  61   CTCTAAATCTTTACCGTCAATAACTCAATCAAAAAACTTTCAACAACGGATCTCTTGGTT  120
            ||||||||||| ||| |||||||| |||| ||||||||||||||||||||||||||||||
Sbjct  77   CTCTAAATCTTAACCCTCAATAACACAATTAAAAAACTTTCAACAACGGATCTCTTGGTT  136

Query  121  CTCGCATCGATGAAGAACGCAGCGAATTGCGATACGTAATATGACTTGCAGACGTGAATC  180
            ||||||||||||||||||||||||||||||||||||||||||||||||||||||||||||
Sbjct  137  CTCGCATCGATGAAGAACGCAGCGAATTGCGATACGTAATATGACTTGCAGACGTGAATC  196

Query  181  ATTGAATCTTTGAACGCACATTGCGCCCCGGGGTATTCCCCAGGGCATGCGTGGGTGAGC  240
            ||||||||||||||||||||||||||||||||||||||||||||||||||||||||||||
Sbjct  197  ATTGAATCTTTGAACGCACATTGCGCCCCGGGGTATTCCCCAGGGCATGCGTGGGTGAGC  256

Query  241  GATATTTACTCTCAAACCTCCGGTTTGGTCCTGCTTCGGCCTAATATCAACGGCGCTAGA  300
            ||||||||||||||||||||||||||||||||||||||||||||||||||||||||||||
Sbjct  257  GATATTTACTCTCAAACCTCCGGTTTGGTCCTGCTTCGGCCTAATATCAACGGCGCTAGA  316

Query  301  ATAAGTTTTAGCCCCATTCTTCTTCCTCACCCTCGTAAGACTACCCGCTGAACTTAAGCA  360
            ||||||||||||||||||||||||||||||||||||||||| ||||||||||||||||||
Sbjct  317  ATAAGTTTTAGCCCCATTCTTCTTCCTCACCCTCGTAAGACAACCCGCTGAACTTAAGCA  376

Query  361  TATCAATAAGCGGAGGA  377
            ||||| |||||||||||
Sbjct  377  TATCATTAAGCGGAGGA  393
```
