# Supplementary material for: Isolation of local strains of the yeast Metschnikowia for biocontrol and lipid production purposes
Source: World J Microbiol Biotechnol. 2024 Feb 9;40(3):88. doi: 10.1007/s11274-024-03918-y (PMC10857958; doi:10.1007/s11274-024-03918-y)
Supplement: Supplementary file 3 — Supplementary Material 3 [file 11274_2024_3918_MOESM3_ESM.pdf]

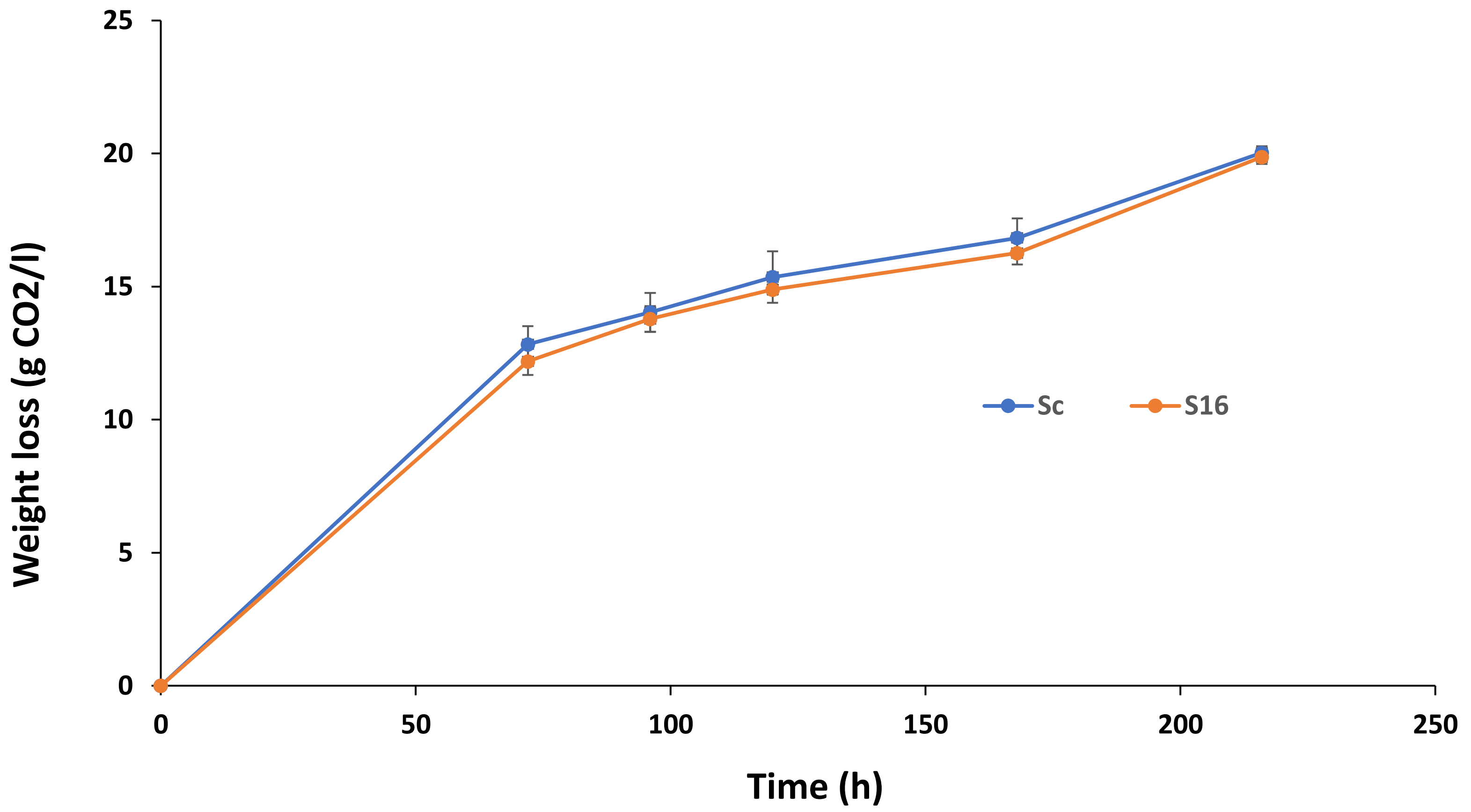

Supplementary Figure 2. Weight loss during a fermentation in potato hydrolisate by a *S. cerevisiae* strain and *M. fructicola* S16 strain.
